# ANGPTL2 inhibits macrophage pyroptosis and alleviates rheumatoid arthritis progression by regulating mitophagy via IGFBP5

**DOI:** 10.1038/s41419-026-08537-z

**Published:** 2026-03-12

**Authors:** Yuqi Liu, Qiudong Yang, Zhendong Huang, Jiahui Sun, Junhong Xiao, Zhengkun Yang, Xin Huang, Li Ma, Xiaoxuan Wang, Chuan Wang, Zhengguo Cao

**Affiliations:** 1 State Key Laboratory of Oral & Maxillofacial Reconstruction and Regeneration, Key Laboratory of Oral Biomedicine, Ministry of Education, Hubei Key Laboratory of Stomatology, School & Hospital of Stomatology, Wuhan University, Wuhan, China; 2 Department of Periodontology, School & Hospital of Stomatology, Wuhan University, Wuhan, China

**Keywords:** Rheumatoid arthritis, Cell death and immune response, Mitophagy

## Abstract

Dysregulated macrophage pyroptosis and impaired mitophagy have emerged as critical drivers of rheumatoid arthritis (RA) progression, yet their upstream regulatory mechanisms remain unclear. Previous studies have demonstrated that ANGPTL2 deficiency aggravates alveolar bone loss in periodontitis, a condition that shares mechanistic similarities with RA in terms of bone destruction. Given the established link between periodontitis and RA, these findings suggest that ANGPTL2 may also play a protective role in RA-related joint pathology. In this study, we demonstrate that ANGPTL2 deficiency worsens joint inflammation, bone erosion, and macrophage pyroptosis in mice with collagen-induced arthritis (CIA). Mechanistically, ANGPTL2 loss impairs mitophagy and promotes mitochondrial dysfunction by inhibiting IGFBP5, leading to sustained NLRP3 inflammasome activation. Intra-articular administration of AAV-*Angptl2* restores mitophagy, suppresses pyroptosis, and alleviates RA pathology. These findings identify ANGPTL2 as a key regulator of macrophage mitophagy and suggest its therapeutic potential in RA.

**Figure:**
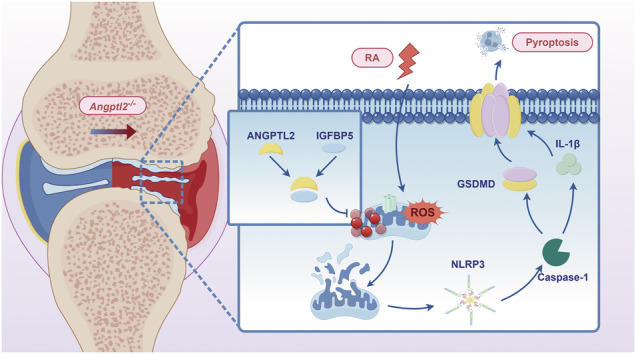
Schematic diagram of the mechanism of ANGPTL2 in the treatment of rheumatoid arthritis.

Schematic diagram of the mechanism of ANGPTL2 in the treatment of rheumatoid arthritis.

## Introduction

Rheumatoid arthritis (RA) is a chronic autoimmune disease characterized by persistent synovial inflammation, cartilage degradation, and bone erosion, in which macrophage-driven inflammation plays a central pathogenic role [1, 2]. Pyroptosis, a pro-inflammatory form of programmed cell death mediated primarily by the NOD-like receptor family pyrin domain-containing 3 (NLRP3) inflammasome, contributes to joint inflammation and structural damage [3]. Emerging evidence links mitochondrial dysfunction and defective mitophagy to excessive inflammasome activation and pyroptotic cell death in RA [4]. Angiopoietin-like 2 (ANGPTL2), a multifunctional protein involved in immune regulation and tissue remodeling, has been implicated in various inflammatory and metabolic disorders [5], but its role in RA and its potential regulation of macrophage mitophagy remain unclear. This study aimed to investigate the function and mechanism of ANGPTL2 in regulating macrophage pyroptosis via mitophagy and to evaluate its therapeutic potential in RA.

RA is a systemic autoimmune disease characterized by chronic synovial inflammation that progressively damages the joints, resulting in swelling, pain, cartilage degradation, bone erosion, and eventual functional disability [1, 2]. Beyond its significant impact on quality of life, RA can also cause widespread multi-organ involvement [6–8]. Current therapeutic regimens—including nonsteroidal anti-inflammatory drugs (NSAIDs), disease-modifying antirheumatic drugs (DMARDs), and biologic agents [9]—have improved disease control but remain insufficient to fully prevent inflammation-driven bone destruction. Persistent joint inflammation and progressive bone loss continue to pose major clinical challenges [10]. This underscores the urgent need to explore new therapeutic targets and develop more effective strategies to halt disease progression.

ANGPTL2 is a multifunctional protein involved in angiogenesis, metabolic regulation, and immune modulation [5]. It has been implicated in diverse pathological conditions, including cardiovascular disease, diabetes, and cancer [11–13]. Yet its involvement in bone-resorptive disorders remains poorly characterized. Our previous work demonstrated that ANGPTL2 is critical in periodontitis, where its deficiency exacerbates bone loss, highlighting its significant role in bone metabolism [14]. Given the well-established association between periodontitis and RA—both of which share overlapping pathogenic mechanisms such as inflammatory bone destruction [15]—ANGPTL2 may also influence the progression of arthritis. Although some studies have reported on ANGPTL2’s involvement in bone-destroying diseases [16, 17], its specific role in RA, particularly in regulating macrophage function and modulating disease progression, remains largely unknown.

Immune cells, particularly macrophages, are key mediators in the pathogenesis of RA, driving both persistent inflammation and bone destruction [18, 19]. In the synovial microenvironment, activated macrophages release large amounts of pro-inflammatory cytokines, which are central to sustaining joint inflammation [20]. Pyroptosis, an intensely inflammatory form of programmed cell death, plays an important role in innate immunity and chronic inflammatory disorders [21, 22]. In macrophages, pyroptosis is initiated predominantly by activation of canonical inflammasomes, which triggers Caspase-1–dependent cleavage of gasdermin D (GSDMD) and formation of membrane pores [22, 23]. This results in the release of inflammatory cytokines such as interleukin (IL)-1β and IL-18, amplifying local inflammatory responses [23]. Among the various inflammasome complexes, the NLRP3 inflammasome is a well-characterized sensor that integrates diverse danger signals to initiate pyroptotic signaling. Pyroptosis of macrophages contributes to a pro-inflammatory microenvironment in the joint, promoting immune cell infiltration and perpetuating joint destruction [24, 25]. Therefore, regulation of macrophage pyroptosis—particularly through modulation of NLRP3 inflammasome activity—is considered critical in the development and progression of RA. Recent studies have highlighted the central role of the NLRP3 inflammasome in RA, with its excessive activation contributing significantly to the inflammatory cascade [26]. However, despite the established importance of the NLRP3 inflammasome in RA, the regulatory mechanisms controlling its activation are not yet fully understood, particularly in terms of how to effectively modulate its activity to slow disease progression.

Autophagy is a fundamental intracellular degradation process that preserves cellular homeostasis by eliminating damaged organelles and misfolded proteins through lysosomal recycling [27]. Mitophagy, a selective form of autophagy, specifically targets dysfunctional mitochondria for degradation [28], serving as a vital quality control mechanism to maintain mitochondrial integrity and suppress oxidative stress [29]. Impaired mitophagy results in the accumulation of damaged mitochondria and excessive production of reactive oxygen species (ROS), which can trigger NLRP3 inflammasome activation and amplify pyroptotic cell death [30, 31]. In RA, disturbances in autophagic processes—including defective mitophagy—have been linked to heightened synovial inflammation, cartilage damage, and bone erosion [32, 33]. These findings highlight a complex crosstalk between mitophagy and pyroptosis in macrophages, suggesting that mitochondrial homeostasis plays a key role in restraining inflammatory responses during RA progression.

Therefore, this study aims to elucidate the mechanisms of joint damage progression in RA, focusing on ANGPTL2-mediated mitophagy in macrophages and its role in alleviating pyroptosis. Mechanistically, ANGPTL2 directly interacts with IGFBP5 to regulate mitophagy, consequently suppressing NLRP3 inflammasome activation and attenuating macrophage pyroptosis. Overall, our findings not only provide new insights into the role of ANGPTL2 in immune-inflammatory processes but also identify potential therapeutic targets for controlling both inflammation and bone resorption in RA, with important clinical implications.

## Results

### ANGPTL2 deficiency exacerbates pyroptosis and joint damage in a mouse model of rheumatoid arthritis

**Fig. 1:**
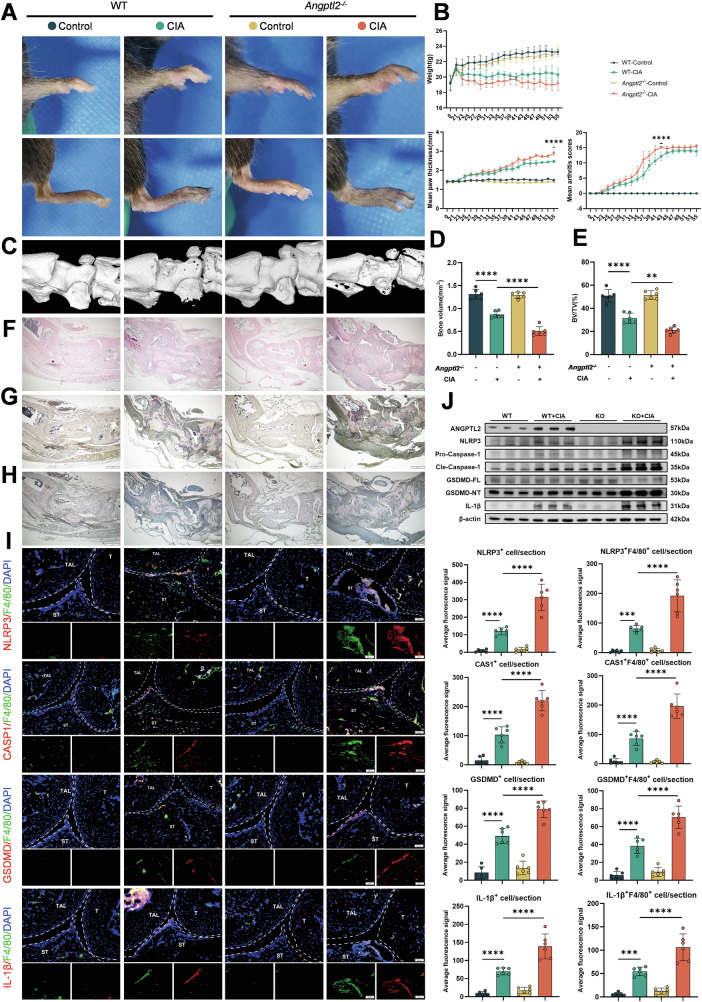
ANGPTL2 deficiency exacerbates pyroptosis and joint damage in a mouse model of rheumatoid arthritis. **A** Representative macroscopic images showing paw swelling in WT and *Angptl2*^*–/–*^ CIA mice. **B** Assessment of arthritis progression, including body weight changes, paw thickness, and clinical arthritis scores over time. **C** Representative 3D μCT reconstruction images of ankle joints. Quantification of talus bone volume (mm³) (**D**) and the ratio of bone volume to tissue volume (BV/TV, %) (**E**) in ankle joints. **F** Representative H&E-stained sections of ankle joints showing inflammation and structural damage. **G** Representative TRAP-stained sections of ankle joints showing osteoclast distribution and activity. **H** Representative ALP-stained sections of ankle joints indicating osteoblast activity and bone formation in periarticular regions. **I** Dual immunofluorescence (IF) staining of ankle joint sections using anti-F4/80 and antibodies against NLRP3, Caspase-1, GSDMD, or IL-1β in CIA mice. The average IF signals for total NLRP3, Caspase-1, GSDMD, and IL-1β expression, and co-localization with F4/80^+^ macrophages were determined. T: tibia; TAL: talus; ST: synovial tissue. **J** Western blot analysis showing protein levels of ANGPTL2, NLRP3, Caspase-1, GSDMD, and IL-1β in the joints of CIA mice. *p < 0.05, **p < 0.01, ***p <0.001, ****p <0.0001 in the indicated groups.

The role of ANGPTL2 in RA was investigated using a collagen-induced arthritis (CIA) model in wild-type (WT) and ANGPTL2-deficient (*Angptl*2^–/–^) mice (Fig. S1A). Compared to WT controls, *Angptl2*^*–/–*^ mice exhibited significantly greater arthritis severity, as reflected by increased paw swelling, elevated clinical arthritis scores, and reduced body weight over time (Fig. 1A, B). Micro-CT revealed severe bone erosion in *Angptl2*^*–/–*^ mice, with reduced talus bone volume and a markedly lower bone volume/tissue volume (BV/TV) ratio compared to WT mice (Fig. 1C–E).

Histological assessment further supported these findings. Hematoxylin and eosin (H&E) staining showed increased synovial hyperplasia, inflammatory infiltration, and cartilage erosion in *Angptl2*^*–/–*^ joints (Fig. 1F). Tartrate-resistant acid phosphatase (TRAP) staining showed enhanced osteoclast presence and activity in the subchondral bone area of *Angptl2*^*–/–*^ mice (Fig. 1G), whereas alkaline phosphatase (ALP) staining indicated reduced osteoblast activity in periarticular regions (Fig. 1H), suggesting a more severe imbalance in bone remodeling toward net bone loss in the *Angptl2*^*–/–*^ group.

To assess pyroptotic activity in joint-resident macrophages, dual immunofluorescence staining for F4/80 and pyroptosis-related markers (NLRP3, GSDMD, Caspase-1, IL-1β) was performed. Quantitative analysis revealed that both the total expression levels of NLRP3, GSDMD, Caspase-1, and IL-1β and their co-localization with F4/80^+^ macrophages were significantly increased in *Angptl2*^*–/–*^ mice (Fig. 1I). These findings indicate that in macrophages lacking ANGPTL2, the activation of the inflammasome is enhanced and pyroptosis increases.

At the molecular level, qPCR analysis of joint tissues confirmed that *Angptl2* expression was almost undetectable in *Angptl2*^*–/–*^ mice (Fig. S1B). Furthermore, relative to WT RA mice, *Angptl2*^*–/–*^ RA mice exhibited upregulated expression of pyroptosis-associated genes, including *Nlrp3*, *Gsdmd*, *Caspase-1*, and *Il-1β* (Fig. S1C). Consistently, Western blot analysis demonstrated elevated protein levels of NLRP3, GSDMD, Caspase-1, and IL-1β in the joints of *Angptl2*^*–/–*^ mice, whereas ANGPTL2 itself was not detected, as expected (Fig. 1J). Collectively, these findings indicate that ANGPTL2 deficiency aggravates inflammation and bone damage in RA, potentially through increased activation of the NLRP3 inflammasome and pyroptosis signaling pathways.

### Inflammatory stimulation reduces ANGPTL2 expression and induces pyroptotic signaling in macrophages in vitro

**Fig. 2:**
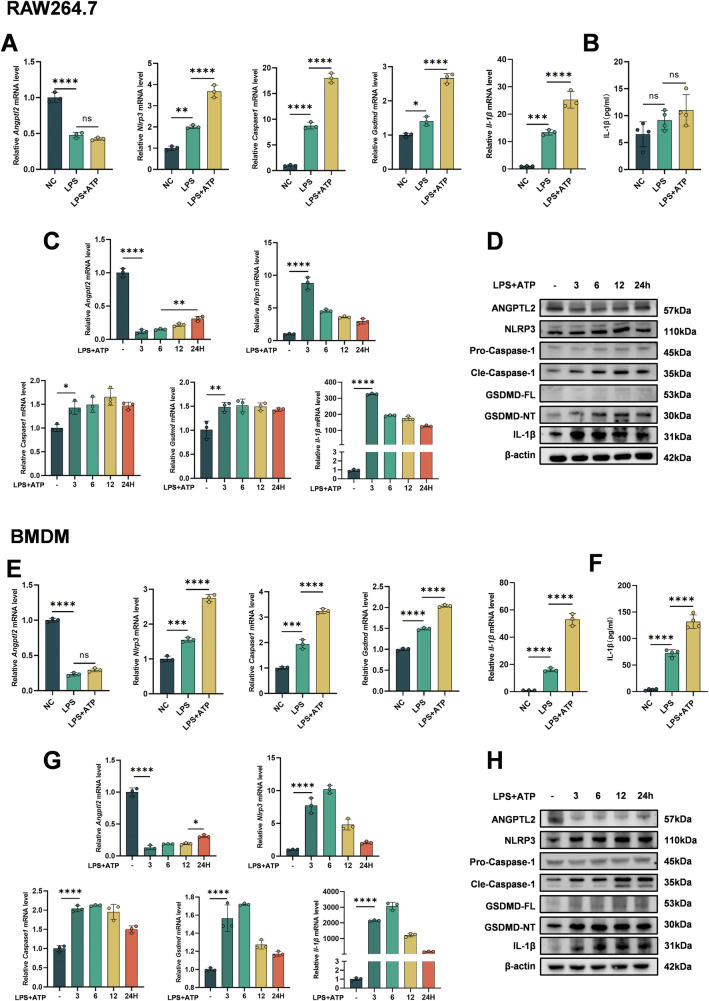
Inflammatory stimulation reduces ANGPTL2 expression and induces pyroptotic signaling in macrophages in vitro. **A** qPCR analysis of *Angptl2*, *Nlrp3*, *Caspase-1*, *Gsdmd*, and *Il-1β* expression in RAW264.7 cells stimulated with LPS alone or LPS + ATP. **B** ELISA quantification of IL-1β secretion in culture supernatants from RAW264.7 cells stimulated with LPS alone or LPS + ATP. **C** Time-course qPCR analysis of *Angptl2*, *Nlrp3*, *Caspase-1*, *Gsdmd*, and *Il-1β* mRNA expression in RAW264.7 cells primed with LPS for 0, 3, 6, 12, and 24 h, followed by 1-h ATP stimulation prior to RNA extraction. **D** Corresponding time-course Western blot analysis of ANGPTL2, NLRP3, GSDMD, Caspase-1, and IL-1β expression in RAW264.7 cells under identical conditions. **E** qPCR analysis of *Angptl2*, *Nlrp3*, *Caspase-1*, *Gsdmd*, and *Il-1β* expression in BMDMs stimulated with LPS alone or LPS + ATP. **F** ELISA quantification of IL-1β secretion in culture supernatants from BMDMs stimulated with LPS alone or LPS + ATP. **G** Time-course qPCR analysis of *Angptl2*, *Nlrp3*, *Caspase-1*, *Gsdmd*, and *Il-1β* mRNA expression in BMDMs primed with LPS for 0, 3, 6, 12, and 24 h, followed by 1-h ATP stimulation prior to RNA extraction. **H** Corresponding time-course Western blot analysis of ANGPTL2, NLRP3, GSDMD, Caspase-1, and IL-1β expression in BMDMs under identical conditions. *p < 0.05, **p < 0.01, ***p <0.001, ****p <0.0001 in the indicated groups.

To further validate these findings in vitro, RAW264.7 cells and Bone marrow–derived macrophages (BMDMs) were stimulated with LPS or LPS combined with ATP to assess the effect of inflammatory signals on ANGPTL2 expression and inflammasome activation [34]. qPCR results showed that *Angptl2* expression was downregulated upon stimulation in both cells (Fig. 2A, E). Upon LPS treatment alone, the expression of pyroptosis-related genes (*Nlrp3*, *Caspase-1*, *Gsdmd*, *Il-1β*) was moderately upregulated. In contrast, the combination of LPS priming followed by ATP stimulation resulted in a robust and synergistic induction of these genes, indicative of full activation of the inflammasome cascade (Fig. 2A, E). ELISA results of BMDMs were consistent with transcriptional trends, showing a marked increase in IL-1β secretion upon LPS + ATP treatment compared to LPS alone (Fig. 2F). RAW264.7 cells failed to release mature IL-1β (Fig. 2B), consistent with previous findings that these cells lack ASC and are unable to efficiently process and secrete mature IL-1β [35]. To further investigate the temporal dynamics of ANGPTL2 expression and inflammasome activation, RAW264.7 cells and BMDMs were primed with LPS for varying durations (0, 3, 6, 12, and 24 h), followed by ATP stimulation for 1 h prior to sample collection. qPCR analysis revealed that *Angptl2* expression dropped sharply immediately after stimulation, followed by a gradual recovery over time (Fig. 2C, G). In contrast, the expression of inflammasome-related genes (*Nlrp3*, *Gsdmd*, *Caspase-1*, and *Il-1β*) exhibited a biphasic response, with transcript levels peaking at 3-6 h and subsequently declining at 12-24 h (Fig. 2C, G). Despite the decrease over time, the expression of these inflammatory mediators remained elevated relative to baseline, indicating that cells maintained a sustained pro-inflammatory state. Western blot analysis confirmed the qPCR trends at the protein level. The ANGPTL2 expression decreased at 3–12 h, slightly rebounded at 24 h but remained below the baseline level, while the expression of NLRP3, GSDMD, Caspase-1, and IL-1β increased markedly during early time points and then showed partial attenuation, yet remained higher than baseline at 24 h (Fig. 2D, H). These results suggest a dynamic inverse relationship between ANGPTL2 expression and pyroptosis-related gene activation under inflammatory conditions, suggesting that it may have a potential regulatory role in inhibiting macrophage cell pyroptosis.

### Loss of ANGPTL2 enhances LPS-induced pyroptosis in macrophages

**Fig. 3:**
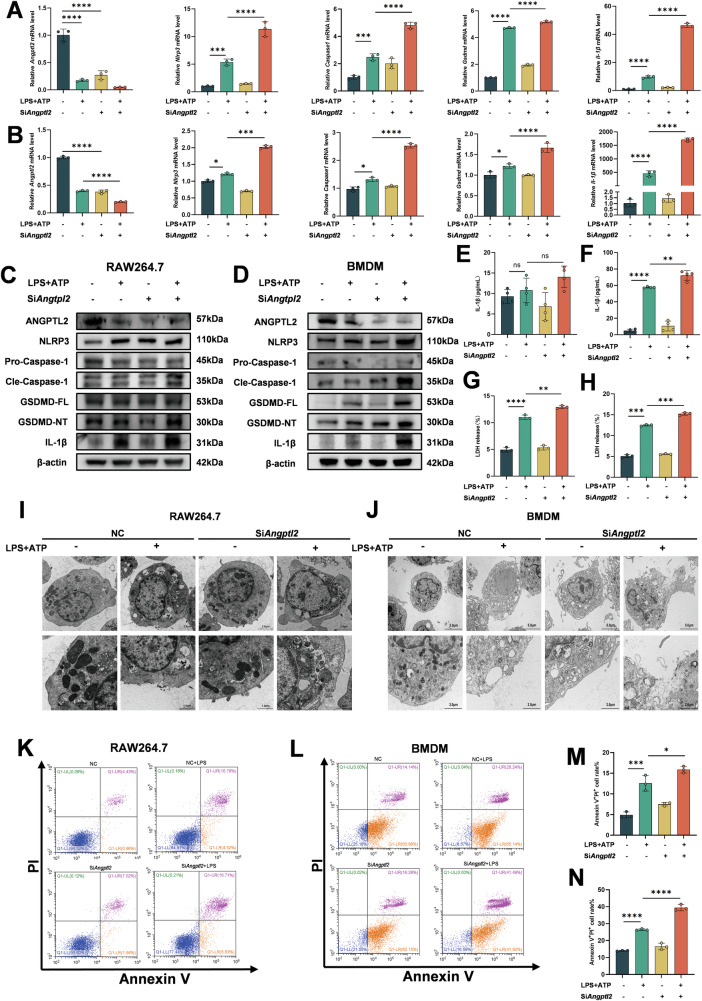
Knockdown of ANGPTL2 enhances LPS-induced pyroptosis in macrophages. qPCR analysis of *Angptl2*, *Nlrp3*, *Caspase-1*, *Gsdmd*, and *Il-1β* mRNA expression in RAW264.7 cells (**A**) and BMDMs (**B**) following si*Angptl2* or siNC transfection, with or without LPS + ATP stimulation. Western blot analysis of ANGPTL2, NLRP3, GSDMD, Caspase-1, and IL-1β protein expression in RAW264.7 cells (**C**) and BMDMs (**D**) under identical conditions. ELISA measurement of IL-1β levels in culture supernatants of RAW264.7 cells (**E**) and BMDMs (**F**). Cell death assessment using LDH release assay in RAW264.7 cells (**G**) and BMDMs (**H**) under identical conditions. Representative TEM images showing morphological features of pyroptotic cell death in RAW264.7 cells (**I**) and BMDMs (**J**). Representative flow cytometry plots showing Annexin V and PI staining of RAW264.7 cells (**K**) and BMDMs (**L**) under identical conditions. Quantification of Annexin V^+^PI^+^ populations in RAW264.7 cells (**M**) and BMDMs (**N**). *p < 0.05, **p < 0.01, ***p < 0.001, ****p < 0.0001 in the indicated groups.

To investigate the function of ANGPTL2 in macrophage pyroptosis, *Angptl*2 expression was silenced in RAW264.7 cells and BMDMs using siRNA. Efficient knockdown of *Angptl2* at both mRNA and protein levels was verified by qPCR and Western blotting in both cell types (Fig. 3A–D).

The expression of pyroptosis-related genes was then assessed. Baseline levels of *Nlrp3*, *Gsdmd*, *Caspase-1*, and *Il-1β* were not significantly affected by *Angptl2* silencing, as determined by qPCR. However, upon stimulation with LPS and ATP, si*Angptl2*-transfected cells displayed significantly higher expression of these genes compared to siNC controls (Fig. 3A, B). Correspondingly, Western blot analysis revealed increased protein levels of NLRP3, GSDMD, Caspase-1, and IL-1β following inflammasome activation in *Angptl2*-deficient cells (Fig. 3C, D). ELISA results further demonstrated a marked increase in IL-1β secretion into the supernatant in BMDMs after *Angptl2* knockdown and stimulation, whereas RAW264.7 cells did not produce detectable levels of mature IL-1β (Fig. 3E, F) [35]. Pyroptosis-associated cell death was evaluated using LDH release assays, which showed enhanced membrane permeability in both RAW264.7 cells and BMDMs transfected with si*Angptl2* following LPS + ATP treatment (Fig. 3G, H). Transmission electron microscopy (TEM) revealed classic features of pyroptosis, such as plasma membrane rupture and organelle swelling, in *Angptl2*-silenced cells upon stimulation (Fig. 3I, J), and quantitative results showed that the percentage of pyroptotic cells increased after silencing ANGPTL2 (Fig. S2G, H). Additionally, flow cytometric analysis using Annexin V and PI staining demonstrated increased proportions of late apoptotic or pyroptotic cells (Annexin V^+^PI^+^) in both cell types after si*Angptl2* treatment (Fig. 3K–N). Fluorescence microscopy with Annexin V/PI staining further confirmed these findings (Fig. S2I, J).

To validate these observations in a genetic model, BMDMs isolated from WT and *Angptl2*^*–/–*^ mice were examined. Following LPS + ATP stimulation, *Angptl2*^*–/–*^ BMDMs displayed increased mRNA expression of *Nlrp3*, *Gsdmd*, *Caspase-1*, and *Il-1β* (Fig. S2A), accompanied by upregulation of the corresponding proteins (Fig. S2B). IL-1β secretion (Fig. S2C), LDH release (Fig. S2D), and Annexin V/PI double-positive staining by flow cytometry (Fig. S2E, F) were all elevated in the knockout group compared to controls, indicating enhanced inflammasome activation and pyroptotic cell death.

These findings collectively indicate that ANGPTL2 serves as a negative regulator of inflammasome-mediated pyroptosis in macrophages and that its deficiency sensitizes cells to inflammatory stimuli, thereby promoting pyroptotic injury.

### Transcriptomic profiling reveals suppression of mitophagy and activation of inflammatory signaling pathways in ANGPTL2-deficient macrophages

**Fig. 4:**
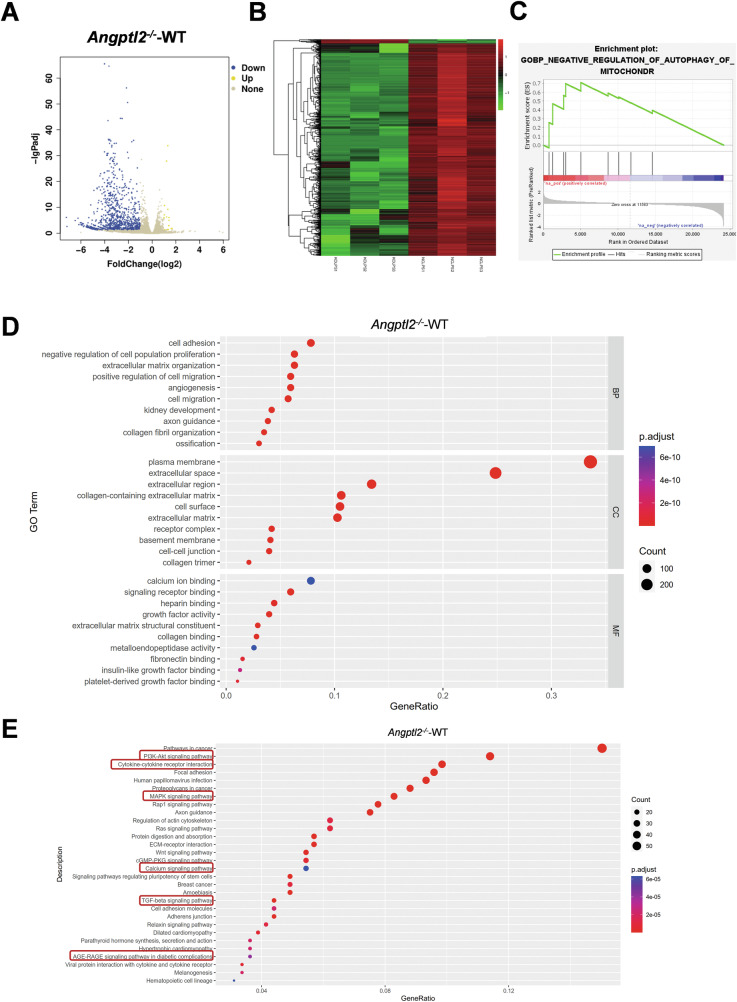
Transcriptomic profiling reveals suppression of mitophagy and activation of inflammatory signaling pathways in ANGPTL2-deficient macrophages. **A** Volcano plot of DEGs comparing *Angptl2*^*–/–*^ and NC BMDMs following LPS + ATP stimulation. Genes with significant upregulation (yellow) or downregulation (blue) are highlighted based on thresholds of adjusted *p* < 0.05 and |log2(fold change)| ≥2. Non-significant genes are shown in gray. **B** Heatmap showing hierarchical clustering of DEGs between *Angptl2*^*–/–*^ and NC BMDMs following LPS + ATP stimulation. Each column represents an individual sample, and each row represents a gene. Color gradient indicates Z-score normalized expression levels (red: upregulated, green: downregulated). **C** GSEA plot showing enrichment of the gene set “negative regulation of autophagy of mitochondrion” between groups. **D** GO enrichment dot plot for DEGs. Enriched terms are classified by Biological Process (BP), Cellular Component (CC), and Molecular Function (MF). Dot size reflects gene count, color indicates adjusted p-value, and X-axis denotes gene ratio. **E** KEGG pathway enrichment map for DEGs in KO-LPS vs. NC-LPS. Dot size reflects gene count, color indicates adjusted p-value, and X-axis denotes gene ratio.

To elucidate the transcriptomic impact of ANGPTL2 deficiency in macrophages, bulk RNA sequencing was conducted on BMDMs derived from *Angptl2*^*–/–*^ and WT mice following LPS priming and subsequent ATP stimulation for 1 h. Analysis identified 876 differentially expressed genes (DEGs) (adjusted *p* < 0.05, |log2(fold change)| ≥2), comprising 17 genes upregulated and 859 genes downregulated in the *Angptl2*^*–/–*^ group relative to WT controls, as depicted in the volcano plot (Fig. 4A). A heatmap of these DEGs demonstrated clear distinctions in gene expression profiles between the two groups, reflecting widespread transcriptomic alterations associated with ANGPTL2 deficiency (Fig. 4B).

Gene Set Enrichment Analysis (GSEA) indicated significant enrichment of the “negative regulation of autophagy of mitochondrion” gene set in *Angptl2*^*–/–*^ BMDMs (Fig. 4C), suggesting a potential suppression of mitophagy-related processes in the absence of ANGPTL2. Gene Ontology (GO) enrichment analysis further showed that DEGs were predominantly involved in extracellular matrix organization, receptor binding, growth factor activity, and structural components of the cell surface (Fig. 4D), pointing to disrupted cell communication and immune interactions. Kyoto Encyclopedia of Genes and Genomes (KEGG) pathway analysis revealed significant enrichment of several signaling pathways in *Angptl2*^*–/–*^ macrophages (Fig. 4E), including multiple inflammation-related cascades such as cytokine-cytokine receptor interaction, TGF-beta, MAPK, PI3K-Akt, and AGE-RAGE signaling. Additionally, pathways associated with mitochondrial function and cellular stress regulation, such as calcium signaling, were also enriched. Collectively, these findings demonstrate that ANGPTL2 deficiency leads to broad alterations in both inflammatory signaling and mitochondrial regulatory pathways in macrophages under inflammatory conditions.

### ANGPTL2 deficiency exacerbates mitochondrial damage and impairs mitophagy in macrophages

**Fig. 5:**
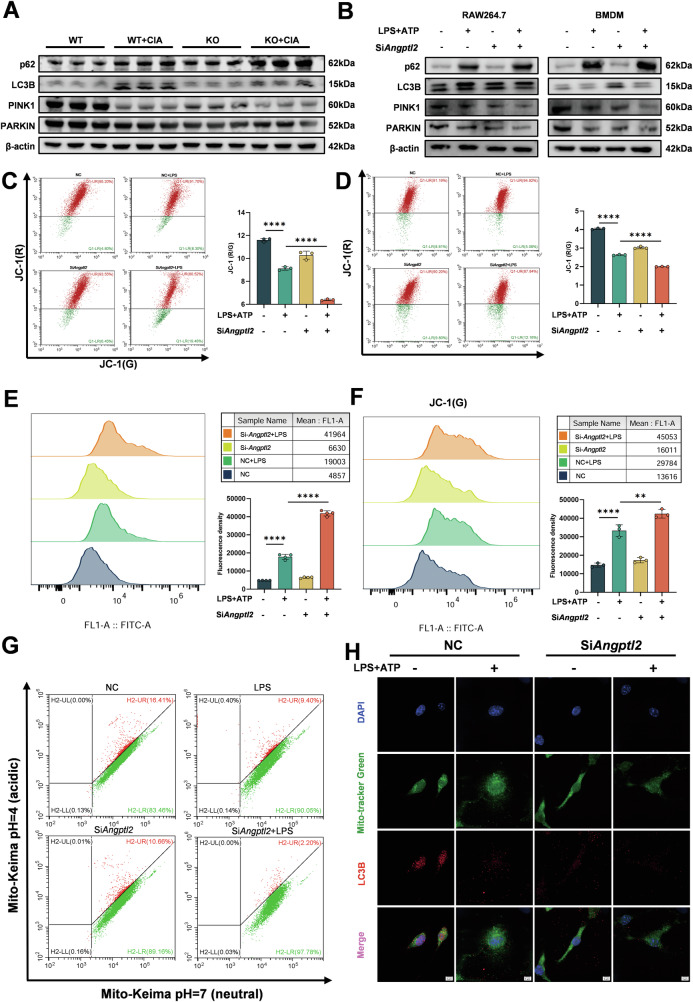
ANGPTL2 deficiency exacerbates mitochondrial damage and impairs mitophagy in macrophages. **A** Western blot analysis of mitophagy-related proteins (PINK1, PARKIN, LC3B, and p62) in joint tissues from WT and *Angptl2*^*–/–*^ mice under basal and CIA-induced conditions. **B** Western blot analysis of mitophagy markers in RAW264.7 cells and BMDMs transfected with si*Angptl2* or siNC, with or without LPS + ATP stimulation. Flow cytometry analysis of mitochondrial membrane potential using JC-1 staining in RAW264.7 cells (**C**) and BMDMs (**D**). The ratio of red (aggregates) to green (monomers) fluorescence (R/G) was calculated to assess mitochondrial integrity. Intracellular ROS levels measured by flow cytometry in RAW264.7 cells (**E**) and BMDMs (**F**) under the indicated conditions. **G** Flow cytometry analysis of mito-Keima-loaded RAW264.7 cells to assess mitophagic flux under different conditions. **H** Representative confocal microscopy images showing colocalization of LC3B (red) and mitochondria (MitoTracker Green) in BMDMs. *p < 0.05, **p < 0.01, ***p < 0.001, ****p < 0.0001 in the indicated groups.

The role of ANGPTL2 in mitochondrial homeostasis was investigated under inflammatory conditions in both in vivo and in vitro models. Western blot analysis of joint tissues from WT and *Angptl2*^*–/–*^ mice showed altered expression of mitophagy-associated proteins, including PINK1, PARKIN, LC3B, and p62 (Fig. 5A). Similarly, in RAW264.7 cells and BMDMs, siRNA-mediated knockdown of *Angptl2* led to marked changes in mitophagy marker expression following LPS + ATP stimulation, indicating disrupted mitophagy (Fig. 5B). Consistent with these results, BMDMs isolated from *Angptl2*^*–/–*^ mice exhibited comparable changes in mitophagy protein levels under the same conditions (Fig. S3A), indicating that ANGPTL2 deficiency impairs mitophagic signaling both at the cellular and tissue levels.

To gain more insight into how ANGPTL2 affects mitochondrial function, the mitochondrial membrane potential was analyzed through JC-1 staining. Flow cytometry revealed a reduced red-to-green fluorescence ratio (R/G) in both RAW264.7 cells and BMDMs after *Angptl2* knockdown and inflammasome activation, indicating enhanced mitochondrial depolarization (Fig. 5C, D). A comparable decrease in membrane potential was also observed in *Angptl2*^*–/–*^ BMDMs compared to WT controls (Fig. S3B). Increased intracellular ROS accumulation was detected in both RAW264.7 cells and BMDMs lacking ANGPTL2 under LPS + ATP stimulation (Fig. 5E, F). This trend was further confirmed in *Angptl2*^*–/–*^ BMDMs (Fig. S3C), suggesting impaired redox balance.

To directly monitor mitophagy activity, RAW264.7 cells were stably transduced with the mito-Keima reporter. Flow cytometry analysis revealed that *Angptl2* knockdown markedly reduced mitophagy, as indicated by a decreased proportion of acidic mito-Keima signals (Figs. 5G and S3D). Consistently, confocal microscopy confirmed this reduction, showing diminished acidic mito-Keima accumulation in ANGPTL2-deficient cells (Fig. S3E, F). Moreover, confocal microscopy analysis showed a decrease in LC3B colocalization with mitochondria in *Angptl2*-deficient BMDMs. LC3B^+^Mitotracker^+^ colocalized signal intensity was reduced (Figs. 5H and S3G), indicating impaired mitophagosome recruitment to damaged mitochondria.

Collectively, these findings demonstrate that ANGPTL2 supports mitochondrial homeostasis and mitophagy under inflammatory conditions, and its deficiency results in mitochondrial dysfunction characterized by membrane depolarization, ROS accumulation, and defective autophagic clearance.

### Recombinant ANGPTL2 restores mitochondrial function and suppresses pyroptosis via mitophagy in BMDMs

**Fig. 6:**
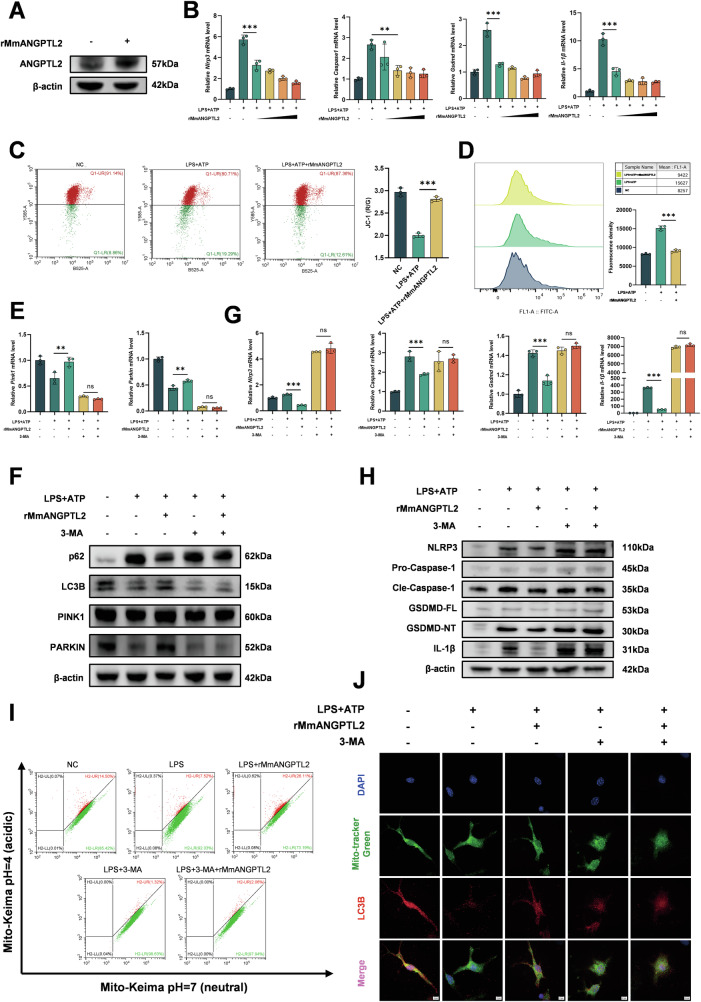
Recombinant ANGPTL2 restores mitochondrial function and suppresses pyroptosis via mitophagy in BMDMs. **A** Western blot analysis of ANGPTL2 expression in BMDMs following treatment with recombinant mouse ANGPTL2 protein (rMmANGPTL2). **B** qPCR analysis of *Nlrp3*, *Caspase-1*, *Gsdmd*, and *Il-1β* mRNA expression in BMDMs stimulated with LPS + ATP and treated with increasing concentrations of rMmANGPTL2 (0, 250, 500, 750, 1000 ng/mL). **C** Flow cytometry analysis of mitochondrial membrane potential using JC-1 staining in BMDMs under NC, LPS + ATP, or LPS + ATP + rMmANGPTL2 treatment conditions. **D** Flow cytometry analysis of intracellular ROS levels in BMDMs under NC, LPS + ATP, or LPS + ATP + rMmANGPTL2 treatment conditions. qPCR (**E**) and Western blot (**F**) analysis of mitophagy-related markers in BMDMs following LPS + ATP stimulation with or without rMmANGPTL2 and the autophagy inhibitor 3-MA. qPCR (**G**) and Western blot (**H**) analysis of pyroptosis-associated markers in BMDMs under the same treatment conditions. **I** Flow cytometry analysis of mito-Keima-loaded RAW264.7 cells to assess mitophagic flux under the same treatment conditions. **J** Representative confocal microscopy images showing LC3B and mitochondria colocalization in BMDMs under the same treatment conditions. *p < 0.05, **p < 0.01, ***p < 0.001, ****p < 0.0001 in the indicated groups.

To examine the impact of ANGPTL2 supplementation on mitochondrial function and inflammasome activation, recombinant mouse ANGPTL2 protein (rMmANGPTL2) was administered to BMDMs stimulated with LPS and ATP. Western blot analysis confirmed increased intracellular ANGPTL2 protein levels following rMmANGPTL2 treatment (Fig. 6A), indicating that the recombinant protein was taken up by cells and exerted its function intracellularly. qPCR results demonstrated that rMmANGPTL2 reduced the expression of inflammasome- and pyroptosis-related genes, including *Nlrp3*, *Caspase-1*, *Gsdmd*, and *Il-1β*, in a dose-dependent manner (Fig. 6B).

JC-1 staining indicated that rMmANGPTL2 restored mitochondrial membrane potential in LPS + ATP-challenged BMDMs (Fig. 6C), while intracellular ROS levels were significantly decreased under the same conditions (Fig. 6D), suggesting improved mitochondrial stability.

To determine whether these protective effects were dependent on mitophagy, the autophagy inhibitor 3-methyladenine (3-MA) was used. rMmANGPTL2 treatment enhanced mitophagy, as evidenced by increased levels of *Pink1* and *Parkin* mRNA expression in qPCR, increased LC3B-II, PINK1 and PARKIN levels and decreased p62 accumulation in Western blot analyses (Fig. 6E, F). However, co-treatment with 3-MA abolished these changes, indicating that ANGPTL2-mediated mitophagy enhancement requires intact autophagic flux.

Notably, the anti-inflammatory and anti-pyroptotic effects of rMmANGPTL2 were also reversed by 3-MA. Inhibition of mitophagy abolished the suppressive effects on LPS + ATP-induced expression of NLRP3, GSDMD, Caspase-1, and IL-1β at both transcriptional and protein levels (Fig. 6G, H), supporting the notion that ANGPTL2 attenuates inflammasome activation through a mitophagy-dependent mechanism. In addition, flow cytometry and confocal microscopy of mito-Keima-loaded RAW264.7 cells confirmed that rMmANGPTL2 restored mitophagy under inflammatory conditions, whereas its protective effect was lost when autophagy was inhibited (Figs. 6I and S4A, B). In parallel, confocal imaging of LC3B and mitochondria in BMDMs revealed that rMmANGPTL2 enhanced LC3B⁺Mitotracker⁺ colocalization, whereas this effect was abolished by 3-MA treatment (Figs. 6J and S4C), further demonstrating that the protective role of ANGPTL2 is mediated through preservation of mitophagosome formation.

Together, these results demonstrate that ANGPTL2 restores mitochondrial function and suppresses pyroptosis in a mitophagy-dependent manner, and this protective effect is abolished upon pharmacological inhibition of autophagy.

### ANGPTL2 modulates mitophagy via an IGFBP5-dependent mechanism

**Fig. 7:**
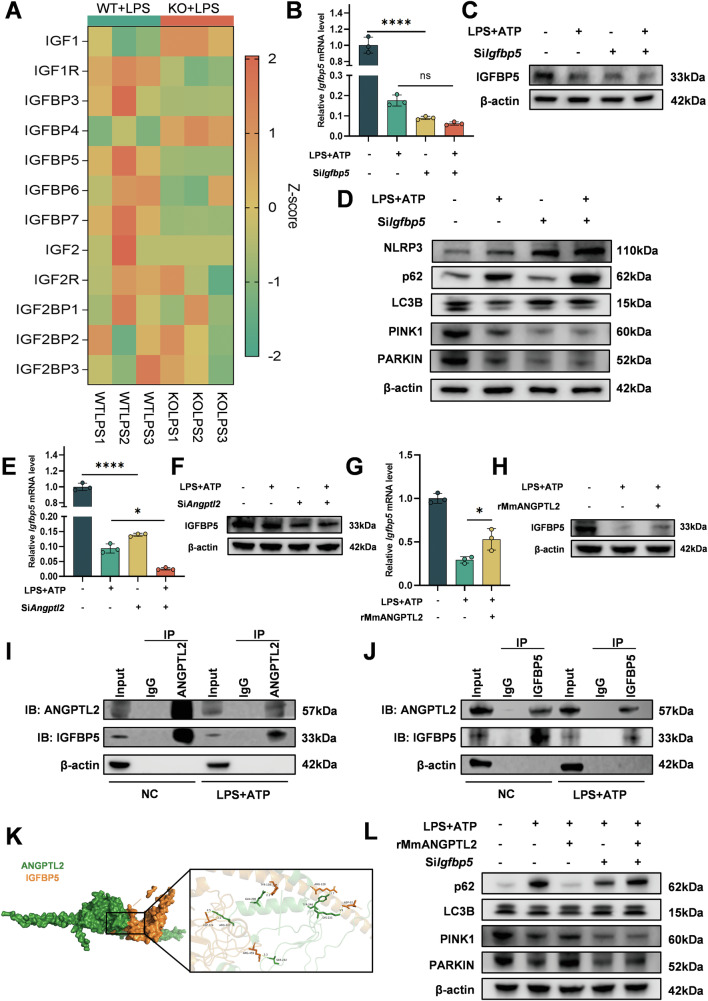
ANGPTL2 modulates mitophagy via an IGFBP5-dependent mechanism. **A** Heatmap showing the expression correlation between ANGPTL2 and insulin-like growth factor (IGF) family members, including IGF1, IGF2, and IGFBP proteins. qPCR (**B**) and Western blot (**C**) analysis of IGFBP5 expression in BMDMs following *Igfbp5* knockdown and LPS + ATP stimulation. **D** Western blot analysis of mitophagy-related proteins (PINK1, PARKIN, LC3B, p62) and NLRP3 in BMDMs after *Igfbp5* knockdown with LPS + ATP treatment. qPCR (**E**) and Western blot (**F**) analysis of IGFBP5 expression in BMDMs following *Angptl2* knockdown and LPS + ATP stimulation. qPCR (**G**) and Western blot (**H**) analysis of IGFBP5 expression in BMDMs treated with rMmANGPTL2 under LPS + ATP stimulation. **I**–**J** Co-immunoprecipitation (Co-IP) assay to evaluate protein-protein interaction between ANGPTL2 and IGFBP5 in RAW264.7 cells. **K** 3D model of structured ANGPTL2 interfaces with IGFBP5 predicted by HDOCK. **L** Western blot analysis of mitophagy-related proteins in BMDMs treated with LPS + ATP and rMmANGPTL2, with or without *Igfbp5* knockdown. *p < 0.05, **p < 0.01, ***p <0.001, ****p <0.0001 in the indicated groups.

To further explore the downstream mediators of ANGPTL2, transcriptomic data from *Angptl2*^*–/–*^ macrophages were analyzed. The results revealed notable alterations in the expression of multiple members of the insulin-like growth factor (IGF) family, including *Igf1*, *Igf2*, and *Igfbp* genes (Fig. 7A), suggesting a potential link between ANGPTL2 and the IGF signaling axis. Among them, the results revealed a marked difference in *Igfbp5* expression between WT and *Angptl2*^*–/–*^ BMDMs (Fig. 7A). Moreover, IGFBP5 has been increasingly recognized in recent studies for its role in regulating autophagy, including mitophagy, in various cell types and disease contexts [36–38]. To further verify this viewpoint, siRNA-mediated knockdown of *Igfbp5* was performed in BMDMs under LPS + ATP stimulation. Efficient reduction of IGFBP5 expression was confirmed by qPCR and Western blotting (Fig. 7B, C). Subsequent analysis revealed that *Igfbp5* knockdown led to impaired expression of mitophagy-related proteins, including PINK1, PARKIN, LC3B, and p62, along with enhanced NLRP3 protein levels (Fig. 7D). Based on these findings, it is reasonable to hypothesize that the regulatory effect of ANGPTL2 on mitophagy is mediated, at least in part, through IGFBP5.

To explore whether IGFBP5 participates in ANGPTL2-mediated mitophagy regulation, the expression of IGFBP5 was assessed in BMDMs following *Angptl2* knockdown or overexpression. Both qPCR and Western blot analysis demonstrated that the knockdown of ANGPTL2 in macrophages markedly decreased IGFBP5 expression (Fig. 7E, F), whereas treatment with recombinant ANGPTL2 significantly restored its expression at both mRNA and protein levels (Fig. 7G, H). Consistent results were observed when ANGPTL2 was overexpressed via lentiviral transduction, with qPCR and Western blot analysis confirming a marked upregulation of IGFBP5 expression (Fig. S5A, B). Co-immunoprecipitation further confirmed a physical interaction between intracellular ANGPTL2 and IGFBP5 proteins (Fig. 7I, J) Immunofluorescence staining also directly observed the colocalization of both proteins within macrophages (Fig. S5C, D), suggesting a direct molecular association. Complementary to these experimental findings, in silico protein-protein docking was conducted using modeled 3D structures of ANGPTL2 and IGFBP5 (Fig. 7K). Docking analysis predicted multiple energetically favorable binding interfaces (docking score = –303.10), supporting the possibility of a functional protein-protein interaction. Furthermore, to validate the functional interplay between these two proteins, BMDMs were treated with rMmANGPTL2 under inflammatory conditions and subsequently transfected with si*Igfbp5*. As expected, ANGPTL2 supplementation restored the expression of mitophagy-related proteins, consistent with previous observations; however, this rescue effect was abolished when IGFBP5 was silenced (Fig. 7L). Comparable results were observed with lentiviral-mediated ANGPTL2 overexpression (Fig. S5E). In addition, Western blot analysis of *Angptl2*^*–/–*^ BMDMs showed that overexpression of IGFBP5 failed to restore mitophagy under LPS + ATP stimulation (Fig. S5F), reinforcing that both ANGPTL2 and IGFBP5 are indispensable components of this regulatory axis.

These findings indicate that under normal conditions, ANGPTL2 maintains mitochondrial homeostasis by upregulating IGFBP5 and forming an intracellular ANGPTL2-IGFBP5 complex that promotes mitophagic signaling. Under inflammatory stress, ANGPTL2 expression is reduced, which diminishes IGFBP5 levels and the complex formation, thereby suppressing PINK1/Parkin activation and leading to mitophagy impairment.

### Intra-articular delivery of ANGPTL2 alleviates rheumatoid arthritis by restoring mitophagy and suppressing pyroptosis

**Fig. 8:**
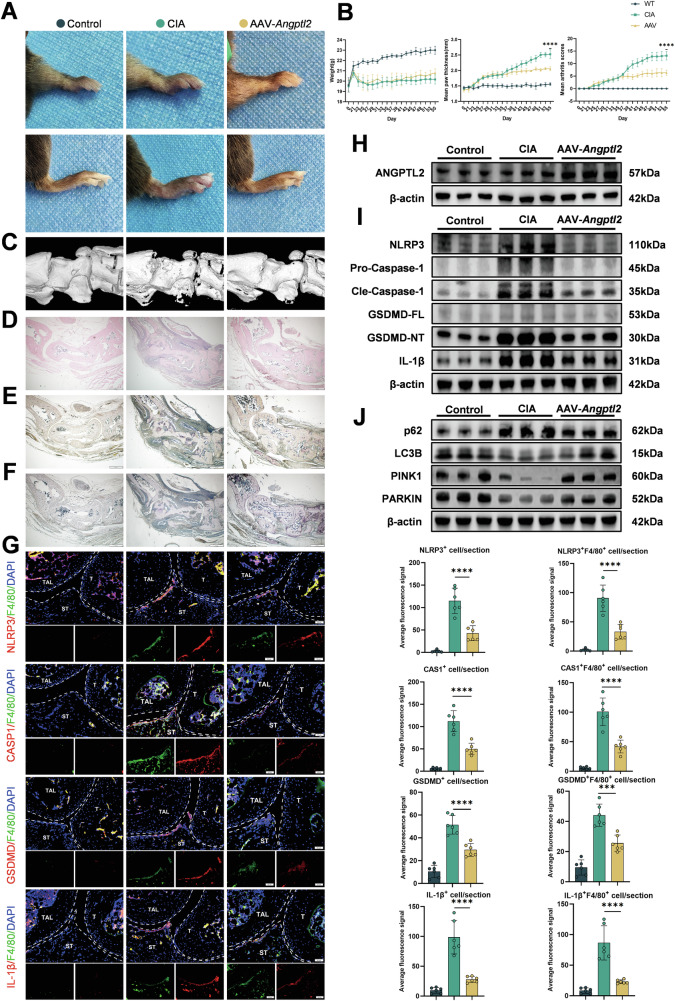
Intra-articular delivery of ANGPTL2 alleviates rheumatoid arthritis by restoring mitophagy and suppressing pyroptosis. **A** Representative images of mouse paws from control, CIA, and AAV-*Angptl2*-treated groups showing macroscopic joint swelling. **B** Assessment of arthritis progression, including body weight changes, paw thickness, and clinical arthritis scores over time. **C** Representative 3D μCT reconstruction images of ankle joints. **D** Representative H&E-stained sections of ankle joints illustrating synovial inflammation and tissue architecture. **E** Representative TRAP-stained sections showing osteoclast distribution and activity. **F** Representative ALP-stained sections indicating osteoblast activity and bone formation. **G** Representative dual immunofluorescence images of ankle joint sections stained for F4/80 and either NLRP3, GSDMD, Caspase-1, or IL-1β. The average IF signals for total NLRP3, GSDMD, Caspase-1, and IL-1β expression, and co-localization with F4/80^+^ macrophages were determined. T: tibia; TAL: talus; ST: Synovial Tissue. **H** Western blot analysis of ANGPTL2 protein levels in joint tissues from each group. **I** Western blot analysis of pyroptosis and inflammasome-related proteins (NLRP3, GSDMD, Caspase-1, IL-1β) in joint tissues. **J** Western blot analysis of mitophagy-related proteins (PINK1, PARKIN, LC3B, p62) in joint tissues. *p < 0.05, **p < 0.01, ***p < 0.001, ****p < 0.0001 in the indicated groups.

To further verify the therapeutic potential of ANGPTL2 in vivo, an adeno-associated virus encoding mouse *Angptl2* (AAV-*Angptl2*) was constructed for intra-articular administration, and the treatment was applied according to a defined timeline following CIA model induction (Fig. S6A). Elevated *Angptl2* expression in joint tissue after AAV-*Angptl2* treatment was confirmed by qPCR (Fig. S6B) and Western blotting (Fig. 8H).

Macroscopic inspection revealed reduced paw swelling in AAV-*Angptl2*-treated mice compared to untreated CIA controls (Fig. 8A). Clinical progression was also mitigated, as evidenced by improved body weight maintenance, decreased paw thickness, and lower arthritis scores over time (Fig. 8B). Structural preservation of the ankle joint was confirmed by micro-CT imaging, with AAV-*Angptl2*-treated mice showing less bone destruction (Fig. 8C). Quantitative micro-CT analysis further demonstrated increased talus bone volume and BV/TV in the treated group (Fig. S6C).

Histological examination supported these findings. H&E staining showed reduced synovial hyperplasia, inflammatory cell infiltration, and bone destruction following AAV-*Angptl2* treatment (Fig. 8D). TRAP and ALP staining indicated a shift in bone remodeling: osteoclast activity was suppressed (Fig. 8E), while osteoblast activity was enhanced (Fig. 8F), suggesting a partial restoration of bone homeostasis in inflamed joints.

To further investigate the effects of AAV-*Angptl2* treatment on cellular inflammation and pyroptosis in vivo, dual immunofluorescence staining of ankle joints was performed. Expression of NLRP3, GSDMD, Caspase-1, and IL-1β was markedly reduced in F4/80^+^ macrophages of AAV-*Angptl2*-treated mice (Fig. 8G). qPCR and Western blot analysis yielded consistent results, showing that the expression of key inflammasome and pyroptosis markers (*Nlrp3*, *Caspase-1*, *Gsdmd*, *Il-1β*) was significantly reduced following AAV-*Angptl2* administration (Figs. S6D and 8I), indicating that ANGPTL2 suppresses inflammation-associated pyroptotic signaling in the joint.

Importantly, mitochondrial quality control was also restored by AAV-*Angptl2* delivery in vivo. Western blot analysis demonstrated increased levels of mitophagy-related proteins, including PINK1, PARKIN, and LC3B-II, along with decreased p62 accumulation (Fig. 8J), suggesting a reactivation of mitophagic flux under inflammatory conditions.

Overall, these results demonstrate that local supplementation of ANGPTL2 via intra-articular gene delivery alleviates joint inflammation and bone erosion, restores mitophagy, and suppresses inflammasome-mediated pyroptosis in CIA mice. These findings highlight ANGPTL2 as a critical regulator of mitochondrial homeostasis and inflammatory resolution in arthritis pathogenesis.

## Discussion

This study identifies ANGPTL2 as a previously unrecognized regulator of mitophagy and inflammatory cell death in RA. Using both murine models and in vitro systems, ANGPTL2 was shown to preserve mitochondrial integrity, enhance mitophagy, and suppress NLRP3 inflammasome-mediated pyroptosis. ANGPTL2 deficiency resulted in mitochondrial dysfunction, excessive ROS, impaired mitophagic flux, and persistent pyroptotic activity, collectively exacerbating synovial inflammation and bone erosion in CIA mice. Importantly, therapeutic delivery of ANGPTL2 into affected joints restored mitochondrial function, mitigated inflammation, and protected against joint destruction. Most notably, this study reveals a mechanistic ANGPTL2-IGFBP5-mitophagy regulatory axis, wherein ANGPTL2 upregulates IGFBP5 expression to maintain mitochondrial homeostasis and inhibit pyroptosis. These findings suggest that targeting this pathway may provide a promising new therapeutic strategy for RA.

While previous studies have generally characterized ANGPTL2 as a pro-inflammatory factor, particularly in metabolic and vascular conditions [39, 40], the current findings offer a distinct perspective by revealing a protective, anti-inflammatory role for ANGPTL2 in macrophage-mediated arthritis. This divergence may be attributed to differences in cellular context and signaling environment. In highly metabolic tissues such as adipose or vasculature, ANGPTL2 appears to amplify stress-induced inflammatory pathways, whereas in macrophage-rich microenvironments such as inflamed joints, ANGPTL2 may stabilize mitochondrial function and enhance mitophagy, thereby restraining excessive NLRP3 inflammasome activation and pyroptosis. These observations suggest that ANGPTL2 functions as a context-dependent regulator whose net effect depends on the balance between pro-inflammatory and homeostatic signaling pathways. Notably, the role of ANGPTL2 in bone-related inflammatory diseases has been largely overlooked. Our previous work demonstrated that ANGPTL2 deficiency exacerbates alveolar bone loss in experimental periodontitis, suggesting a broader function for ANGPTL2 in maintaining bone homeostasis under inflammatory conditions [14]. These data, together with the present findings in CIA mice, support a generalizable model in which ANGPTL2 protects skeletal tissues by regulating both immune responses and mitochondrial quality.

Pyroptosis is increasingly recognized as a critical driver of the inflammatory environment in RA, primarily mediated by aberrant activation of the NLRP3 inflammasome in macrophages [41, 42]. This form of inflammatory cell death amplifies synovial inflammation by promoting the release of IL-1β and IL-18, thereby accelerating cartilage degradation and bone erosion [43]. In the present study, ANGPTL2 deficiency markedly intensified pyroptotic responses both in vivo and in vitro. In CIA mice, loss of ANGPTL2 was associated with elevated expression of NLRP3, Caspase-1, GSDMD, and IL-1β in joint tissues. Consistently, *Angptl2* knockdown in BMDMs and RAW264.7 cells significantly enhanced LPS + ATP-induced inflammasome activation, GSDMD cleavage, and IL-1β secretion, accompanied by increased membrane disruption and cell death. These findings indicate that ANGPTL2 functions as a negative regulator of macrophage pyroptosis, and its absence promotes persistent inflammasome activation. Given the established role of pyroptosis in RA pathogenesis, identifying the upstream regulatory mechanisms of inflammasome activation becomes essential. Notably, mitochondrial dysfunction has been recognized as a potent trigger of pyroptosis [44], suggesting that mitochondrial quality control, particularly mitophagy, may play a central role in the regulatory effects of ANGPTL2.

Mitochondrial dysfunction is a well-established driver of innate immune activation, particularly in the context of RA, where persistent mitochondrial stress contributes to exaggerated inflammasome signaling and sustained production of proinflammatory cytokines [45–47]. Damaged mitochondria release ROS and mitochondrial DNA (mtDNA), both serving as potent danger-associated molecular patterns (DAMPs) that initiate the activation of the NLRP3 inflammasome [48, 49]. The cytosolic release of these mitochondrial byproducts initiates inflammatory cascades, leading to Caspase-1 activation and subsequent maturation of IL-1β, a key cytokine implicated in RA pathogenesis [50, 51]. In this study, macrophages lacking ANGPTL2 exhibited profound mitochondrial dysfunction, as evidenced by decreased mitochondrial membrane potential, excessive intracellular ROS accumulation, and diminished mitophagy. This disruption in mitochondrial quality control was associated with robust NLRP3 inflammasome activation and elevated secretion of mature IL-1β, highlighting the essential role of ANGPTL2 in preserving mitochondrial integrity and limiting inflammasome-mediated inflammation. These results are consistent with previous findings that underscore the importance of mitophagy in regulating inflammasome activity and mitigating inflammatory tissue damage. Defective mitophagy has been implicated in several autoimmune diseases, including RA, lupus, and Crohn’s disease, while enhancing mitophagy—pharmacologically or genetically—has been shown to suppress NLRP3 activation and alleviate disease severity in experimental models [52–54]. The current study builds upon this framework by positioning ANGPTL2 as an upstream regulator of mitophagy to restore mitochondrial homeostasis. Importantly, the data suggest that the pro-inflammatory phenotype observed in *Angptl2*^*–/–*^ macrophages is not solely due to elevated ROS production, but also reflects a failure to remove dysfunctional mitochondria, thereby creating a feed-forward loop of inflammation. This sustained mitochondrial stress likely amplifies inflammasome signaling over time. By enhancing mitophagy, ANGPTL2 effectively breaks this pathogenic cycle, providing a potential immunometabolic checkpoint in chronic inflammation.

One of the major mechanistic insights provided by this study is the identification of a functional link between ANGPTL2 and mitochondrial homeostasis via the IGFBP5 signaling axis. While several regulators of mitophagy have been identified, including PINK1, PARKIN, and BNIP3, little is known about the upstream signals that control mitophagic flux under inflammatory stress [55, 56]. In this context, the finding that intracellular ANGPTL2 promotes mitophagy through IGFBP5 provides a critical new dimension to understanding the immunometabolic crosstalk in macrophages. IGFBP5, a secreted member of the insulin-like growth factor-binding protein family, has previously been implicated in cell differentiation, senescence, and fibrosis in multiple tissue types [57]. Its role in autophagy is emerging, with studies reporting its context-dependent modulation of autophagic flux in various cells and tissues [36–38]. However, its involvement in macrophage mitochondrial function and innate immune signaling has not been previously described. Here, transcriptomic analysis suggested a functional relationship between ANGPTL2 and the IGF family, and further experiments confirmed that ANGPTL2 positively regulates IGFBP5 expression. IGFBP5 knockdown abolished the protective effects of ANGPTL2 on mitophagy, and their direct interaction was confirmed by co-immunoprecipitation. Collectively, these data support a mechanistic model in which intracellular ANGPTL2 maintains mitochondrial integrity and facilitates mitophagy within macrophages via upregulation and interaction with IGFBP5, which in turn limits pyroptosis. This ANGPTL2-IGFBP5-mitophagy axis represents a previously unrecognized regulatory mechanism of immune cell metabolism and inflammation resolution.

Although both ANGPTL2 and IGFBP5 are classically defined as secreted proteins, emerging evidence indicates that many secretory proteins can also exert critical intracellular functions under certain physiological or pathological contexts [58–61]. Increasing studies have demonstrated that secreted proteins may undergo intracellular retention within the endoplasmic reticulum-Golgi apparatus or be re-internalized through endocytic pathways [62–65], allowing them to participate in intracellular signaling regulation and organelle homeostasis. Consistent with these findings, our study revealed detectable intracellular ANGPTL2 and IGFBP5 in macrophages under both basal and stimulated conditions, as confirmed by Western blot and immunofluorescence analyses. Moreover, exogenous supplementation with rMmANGPTL2 increased intracellular ANGPTL2 levels, suggesting that secreted ANGPTL2 can be internalized to exert intracellular effects. Functionally, the reduction of intracellular ANGPTL2 protein not only inhibits the transcription and translation of IGFBP5 but also diminishes the formation of the ANGPTL2-IGFBP5 complex, thereby inducing a downregulation of mitophagy.

While this study identifies IGFBP5 as a downstream mediator of ANGPTL2 in regulating mitophagy, the precise molecular mechanism remains to be elucidated. Prior studies provide potential mechanistic clues: ANGPTL2 has been reported to signal through integrins, particularly integrin α5β1, activating downstream FAK-PI3K-Akt and NF-κB pathways [66–69], both of which are closely linked to mitochondrial quality control and inflammatory regulation. Likewise, IGFBP5 can interact with the IGF-I receptor or exert IGF-independent effects via MAPK/ERK and p38 signaling [70–73], pathways also implicated in autophagy regulation. These findings suggest that ANGPTL2 may modulate IGFBP5 expression or activity through such established signaling routes, thereby influencing mitophagy and inflammasome activation. However, it remains uncertain whether IGFBP5 directly regulates the canonical PINK1/Parkin pathway or acts indirectly through alternative intermediates. Previous studies have indicated that IGFBP5 influences autophagy and mitochondrial dynamics under various physiological and pathological conditions [36–38], yet whether these effects are mediated through direct protein interactions or transcriptional/metabolic regulation is unresolved. Given the limitations of the current study, downstream effectors of ANGPTL2 and IGFBP5 were not fully explored. Future work employing mitochondrial ubiquitination assays, PINK1 stabilization analysis, and Parkin translocation studies will be essential to clarify the mechanism by which ANGPTL2 and IGFBP5 regulate mitophagy. Recognizing this gap highlights the need for deeper mechanistic dissection to refine the ANGPTL2-IGFBP5-mitophagy axis and better assess its therapeutic relevance in RA.

Current therapeutic strategies for RA, including methotrexate, corticosteroids, and biologic agents targeting TNF-α or IL-6, have significantly improved disease management but remain limited by incomplete efficacy, systemic immunosuppression, and potential adverse effects during long-term use. Recent studies have demonstrated that pharmacological modulation of mitophagy can alleviate inflammation and joint destruction in RA models [74–78]. For example, the AMPK activator metformin enhances PINK1/PARKIN-dependent mitophagy, thereby attenuating arthritis severity [79]. Similarly, rapamycin, an mTOR inhibitor, has been shown to stimulate autophagic flux, including mitophagy, and exert anti-inflammatory effects in RA [80, 81]. Despite these promising findings, such agents act systemically and influence multiple metabolic and immune pathways, which may lead to off-target effects, systemic immunosuppression, and limited tissue specificity. These shortcomings highlight the need for targeted strategies capable of restoring mitochondrial homeostasis directly within inflamed joint tissues while minimizing systemic exposure. In this context, our study demonstrates that intra-articular administration of AAV-*Angptl2* effectively restores mitophagy-related protein expression, suppresses inflammasome activation and pyroptosis in macrophages, and mitigates synovial inflammation, bone erosion, and structural joint damage in CIA mice. The ability to deliver ANGPTL2 locally via viral vectors provides a targeted therapeutic approach that may reduce systemic immunosuppression and preserve host defense. Nonetheless, clinical translation will require careful evaluation of long-term safety, tissue-specific expression, dosing parameters, and compatibility with current standard-of-care therapies. Further preclinical and translational studies are warranted to determine whether ANGPTL2-based interventions can be integrated into RA treatment regimens to improve therapeutic outcomes.

Finally, the findings of this study are currently limited to murine models. Although RAW264.7 cells and mouse BMDMs were used to elucidate the in vitro mechanisms, no samples from human patients with rheumatoid arthritis were examined. Although these models provide valuable insights into disease mechanisms, species-specific differences may influence the relevance of ANGPTL2-IGFBP5-mitophagy signaling to human RA. To overcome this limitation and strengthen translational significance, future studies should include primary macrophages isolated from the synovial fluid or peripheral blood of RA patients, as well as immunohistochemical or transcriptomic analyses of joint biopsy specimens. Such approaches would allow direct confirmation of the ANGPTL2-IGFBP5 axis in the human disease context and clarify whether modulation of this pathway has therapeutic potential in patients. Moreover, the upstream regulatory mechanisms controlling ANGPTL2 expression in macrophages under inflammatory stress remain to be elucidated. While IGFBP5 has been identified as a key downstream effector, it is not yet clear whether other IGF-related molecules or parallel signaling pathways contribute to ANGPTL2-mediated regulation of mitophagy. Addressing these questions will be essential to fully understand the biological significance and therapeutic potential of this regulatory axis.

## Conclusion

In conclusion, this study provides the first evidence that ANGPTL2 regulates rheumatoid arthritis progression through a previously unrecognized ANGPTL2-IGFBP5-mitophagy signaling axis. The deficiency of ANGPTL2 inhibits mitophagy, enhances mitochondrial dysfunction, and aggravates the activation of inflammasomes, thereby promoting pyroptosis and joint tissue damage. Moreover, the demonstration that local ANGPTL2 supplementation restores mitophagy and limits joint destruction underscores its potential as a promising therapeutic strategy, particularly in overcoming limitations of current RA treatments. Future investigations should validate these findings in human macrophages and clinical samples, elucidate upstream regulators of ANGPTL2, and assess the long-term safety and efficacy of ANGPTL2-based interventions. Collectively, this work not only identifies a novel molecular target but also provides new insight into mitochondrial quality control as a therapeutic entry point in autoimmune arthritis.

## Materials and methods

### Animals

Wild-type (WT) and ANGPTL2 knockout (*Angptl2*^*–/–*^) C57BL/6J mice were obtained from Cyagen Biosciences (Suzhou, China). The mice were kept in a controlled environment with a temperature of 22 ± 1 °C, 50–60% humidity, and a 12-h light/dark cycle, having unlimited access to food and water. All experimental procedures were approved by the Ethics Committee of the School and Hospital of Stomatology, Wuhan University (S07924110I) and conducted in accordance with the ARRIVE 2.0 guidelines [82].

### Induction of rheumatoid arthritis models

As described before, the CIA was established [83–85]. Male WT and *Angptl2*^*–/–*^ littermates (8 to 10 weeks) were randomly assigned to four groups: untreated WT (WT control), untreated *Angptl2*^*–/–*^ (*Angptl2*^*–/–*^ control), CIA-induced WT (WT RA), and CIA-induced *Angptl2*^*–/–*^ (*Angptl2*^*–/–*^ RA), with 6 mice in each group. Chicken type II collagen (10 mg; Chondrex, Washington, USA; Cat. 20011) was dissolved in 2.5 mL of 0.1 M acetic acid to yield a 4 mg/mL solution and incubated overnight at 4 °C in the dark. The following day, the collagen solution was emulsified (1:1, v/v) with complete Freund’s adjuvant (Sigma-Aldrich, Missouri, USA; Cat.F5881) on ice using a tissue homogenizer. Primary immunization was performed by subcutaneous injection (100 μL) approximately 2 cm from the tail base. On day 21, the collagen solution was similarly emulsified with incomplete Freund’s adjuvant (Sigma-Aldrich, Missouri, USA; Cat.F5506) and used for secondary immunization (100 μL) administered subcutaneously ~3 cm from the tail base. Disease progression was monitored by measuring hind paw thickness with calipers to assess edema, and clinical arthritis scores were evaluated every other day in a double-blinded manner from days 21 to 56. The severity and affected area of the claw deformity are classified using a scale of 0 to 4: 0, normal; 1, mild, but definite redness and swelling of the ankle or wrist, or apparent redness and swelling limited to individual digits, regardless of the number of affected digits; 2, moderate redness and swelling of ankle or wrist; 3, severe redness and swelling of the entire paw including digits; 4, maximally inflamed limb with involvement of multiple joints [86–88]. The clinical arthritis scores are calculated by adding up the scores of all four limbs.

### Micro-CT and histology

Hind paw ankle joints were harvested and fixed in 4% paraformaldehyde. After removal of surrounding soft tissues, joints were scanned using a high-resolution μCT system (SkyScan, Bruker, Massachusetts, USA) with the following settings: 100 kV, 200 μA, and 10 μm voxel resolution. Following scanning, specimens were decalcified for 4weeks, dehydrated, embedded in paraffin, and sectioned at 4 μm thickness. Sections were stained with H&E for general histological evaluation. ALP staining was performed to identify osteoblasts, and TRAP staining was used to detect osteoclasts.

### Cell culture

RAW264.7 cells, routinely maintained in our laboratory, were authenticated by short tandem repeat (STR) profiling and confirmed to be free of mycoplasma contamination prior to experimentation. Cells were cultured in DMEM supplemented with 10% fetal bovine serum (FBS) and 1% penicillin–streptomycin. BMDMs were obtained from the femurs and tibias of WT mice. Bone marrow cells were flushed with α-MEM containing 2% FBS, followed by red blood cell lysis. The remaining cells were cultured in α-MEM supplemented with 10% FBS and macrophage colony-stimulating factor (M-CSF; Abclonal, Wuhan, China) for 3 days to induce macrophage differentiation [89]. All cells were maintained at 37 °C in a humidified incubator with 5% CO₂.

### Immunofluorescence staining

For tissue immunofluorescence, paraffin sections were deparaffinized with xylene, rehydrated through a graded ethanol series, and subjected to antigen retrieval in citrate buffer (pH 6.0) at 95 °C for 20 min before being cooled to room temperature. Sections were permeabilized with 0.2% Triton X-100 for 15 min and blocked with 5% bovine serum albumin (BSA) for 1 h. Primary antibodies against F4/80 (Abcam, Cambridge, UK; ab16911), NLRP3 (Sigma-Aldrich, Missouri, USA; SAB5700723), Caspase-1 (Abmart, Shanghai, China; MU167952), GSDMD (Abclonal, Wuhan, China; A18281), and IL-1β (R&D Systems, Minnesota, USA; AF-401-NA) were applied overnight at 4 °C. After PBS washes, sections were incubated with fluorophore-conjugated secondary antibodies for 1 h at room temperature in the dark. Slides were mounted with DAPI-containing mounting medium (ZhongShan Jinqiao Biotechnology, Beijing, China), and fluorescence images were acquired using an IXplore SpinSR super-resolution confocal microscope.

For cells immunofluorescence, cells that were cultured were seeded onto glass coverslips in 24-well plates, fixed with 4% paraformaldehyde for 15 min at room temperature, and permeabilized with 0.1% Triton X-100 in PBS for 10 min. Cells were blocked with 5% BSA for 1 h and then incubated overnight at 4 °C with anti-LC3B antibody (Sigma-Aldrich, Missouri, USA; L7543) diluted in the blocking buffer. Following PBS washes, cells were incubated with the corresponding fluorophore-conjugated secondary antibody for 1 h at room temperature in the dark. MitoTracker Green (Beyotime, Shanghai, China; C1048) staining was performed at 37 °C for 45 min in the dark. Coverslips were mounted with DAPI-containing mounting medium (ZhongShan Jinqiao Biotechnology, Beijing, China), and images were captured using an Olympus fluorescence microscope. Image analysis was performed with ImageJ software.

### Quantitative real-time PCR

Total RNA was isolated using TRIzol reagent (Invitrogen, California, USA), and 500 ng of RNA was reverse-transcribed into cDNA using PrimeScript RT Master Mix (Takara, RR036A). Quantitative real-time PCR (RT-qPCR) was performed using ChamQ SYBR qPCR Master Mix-Q311 (Vazyme, Nanjing, China) on LightCycler^®^ 480 Instrument II (Roche, Basel, Switzerland). The amplification protocol consisted of an initial denaturation at 95 °C for 30 s, followed by 40 cycles of denaturation at 95 °C for 5 s and annealing/extension at 60 °C for 30 s. A melt-curve analysis was performed to confirm product specificity. Relative mRNA expression was calculated using the 2^−ΔΔCt^ method, with β-actin as the internal reference. Primer sequences are provided in Table S1.

### Western blotting

Total protein was extracted from cultured cells and mouse joint tissues. For tissue protein extraction, mouse knee joints were collected and homogenized in RIPA lysis buffer (Beyotime, Shanghai, China) containing protease and phosphatase inhibitors, utilizing a tissue homogenizer. The homogenates were then centrifuged at 12,000 rpm for 15 min at 4 °C, and the supernatants were collected as total tissue protein. For cellular protein extraction, 100 μL of RIPA lysis buffer supplemented with protease and phosphatase inhibitors was added to each well of a 6-well plate. Cells were lysed via ultrasonic disruption and subsequently centrifuged at 12,000 rpm for 15 min at 4 °C. Protein concentrations were measured using a BCA protein assay kit (Beyotime, Shanghai, China).

20 μg of protein per sample was separated using 12% SDS-PAGE and then transferred onto PVDF membranes. The membranes were treated with 5% non-fat milk in TBST for an hour at room temperature and then incubated overnight at 4 °C with the following primary antibodies: ANGPTL2 (Proteintech, Wuhan. China; 12316-1-AP), NLRP3 (Sigma-Aldrich, Missouri, USA; SAB5700723), Caspase-1 (Abmart, Shanghai, China; MU167952), GSDMD (Abclonal, Wuhan, China; A18281), IL-1β (Huabio, Hangzhou, China; HA601036), p62 (Cell Signaling Technology, Boston, USA; 5114), LC3B (Sigma-Aldrich, Missouri, USA; L7543), PINK1 (Abclonal, Wuhan. China; A7131), PARKIN (Abclonal, Wuhan. China; A0968), IGFBP5 (Abclonal, Wuhan. China; A12451), and β-actin (Proteintech, Wuhan. China; 66009-1-AP). The antibodies were all diluted with QuickBlock™ western primary antibody diluent (Beyotime, Shanghai, China) at a ratio of 1:1000. The following day, membranes were washed three times with TBST (10 min each) and incubated with HRP-conjugated secondary antibodies for 1 h at room temperature. Protein bands were visualized using an Odyssey LI-COR imaging system.

### Electron microscopy

RAW264.7 cells and BMDMs were gathered and fixed in a solution of 2.5% glutaraldehyde in 0.1 M phosphate buffer (pH 7.4) at 4 °C overnight. Following a phosphate buffer wash, the samples were post-fixed in 1% osmium tetroxide for an hour, dehydrated using a series of graded ethanol concentrations, and embedded in epoxy resin. Using an ultramicrotome, ultrathin sections were created, mounted on copper grids, and stained with uranyl acetate and lead citrate. The ultrastructural characteristics associated with pyroptosis, such as plasma membrane rupture, organelle swelling, and chromatin condensation, were examined using a transmission electron microscope (TEM; Hitachi HT7700, Tokyo, Japan) at an accelerating voltage of 80 kV.

### Lactate dehydrogenase (LDH) release assay

Pyroptotic cell death was evaluated by measuring the release of lactate dehydrogenase (LDH) into the culture supernatant, using an LDH Cytotoxicity Assay Kit (Beyotime, Shanghai, China) according to the manufacturer’s instructions. The level of LDH release served as a marker for plasma membrane rupture associated with pyroptosis. Briefly, RAW264.7 cells and BMDMs were seeded in 96-well plates and treated as indicated. At the end of stimulation, 120 µL of culture supernatant from each well was transferred to a new 96-well plate, and 60 µL of LDH substrate mix was added. The plate was incubated at room temperature in the dark for 30 min. The reaction was terminated by adding 50 µL of stop solution, and absorbance was measured at 490 nm using a microplate reader. Maximum LDH release was determined by treating parallel wells with LDH release reagent to lyse all cells, and spontaneous LDH release was assessed in untreated control wells. The percentage of pyroptosis was calculated as: Cell toxicity or mortality (%) = (Experimental LDH release—Spontaneous LDH release)/(Maximum LDH release—Spontaneous LDH release) × 100.

### ELISA

The supernatant was collected post-cell culture and analyzed via an enzyme-linked immunosorbent assay (ELISA) to assess cytokine levels. Mouse IL-1β was measured with a commercial ELISA kit (Abclonal, Wuhan, China; RK00006) following the manufacturer’s instructions. Briefly, 96-well plates pre-coated with capture antibody were incubated with 100 µL of standards or appropriately diluted samples in duplicate and incubated for 2 h at room temperature. After washing, biotin-conjugated detection antibody was added, followed by streptavidin-HRP. Color development was performed using tetramethylbenzidine (TMB) substrate, and the reaction was stopped with 2 M sulfuric acid. Optical density (OD) was measured at 450 nm with a reference wavelength of 570 nm using a microplate reader.

### Flow cytometry

Flow cytometry was used to evaluate apoptosis, mitochondrial membrane potential, levels of intracellular ROS, and mitophagy. Cell apoptosis was evaluated using an Annexin V-FITC/PI Apoptosis Detection Kit (Yeasen, Shanghai, China). The mitochondrial membrane potential was measured with a JC-1 staining kit (Beyotime, Shanghai, China), while intracellular ROS levels were determined using a ROS assay kit (Beyotime, Shanghai, China). All staining procedures were carried out according to the manufacturer’s instructions.

### Gene knockdown and overexpression

RAW264.7 cells and BMDMs were subjected to gene knockdown or overexpression as indicated. For gene silencing, cells were transfected with siRNA using Lipofectamine 2000 (Invitrogen, California, USA) following the manufacturer’s protocol. For gene overexpression, cells were transiently transfected with an IGFBP5 overexpression vector or its corresponding empty control using the same transfection reagent. Cells were harvested 48–72 h post-transfection for further analyses. The siRNA sequences utilized are provided in Table S2.

### RNA sequencing and data analysis

Total RNA was isolated from BMDMs obtained from WT and *Angptl2*^*–/–*^ mice using TRIzol™ reagent (Invitrogen, California, USA), following the manufacturer’s instructions. Sequencing libraries were constructed with the NEBNext^®^ Ultra™ RNA Library Prep Kit (New England Biolabs, Massachusetts, USA), and paired-end sequencing was conducted on an Illumina NovaSeq 6000 platform (Illumina, California, USA). Raw reads underwent quality control with FastQC and were trimmed using Trimmomatic. Clean reads were aligned to the mouse reference genome (GRCm38/mm10) using HISAT2, and gene expression was quantified with StringTie. DEGs were pinpointed using DESeq2, applying the criteria of |log₂FoldChange|≥2 and an adjusted P-value less than 0.05. Functional enrichment analyses, encompassing GO, KEGG, and GSEA, were conducted with the clusterProfiler R package.

### Mito-Keima mitophagy assay

RAW264.7 cells were stably transduced with a mito-Keima lentiviral construct (HANBIO, Shanghai, China) to monitor mitophagy. Lentiviral particles were produced and used to infect RAW264.7 cells according to the manufacturer’s instructions, followed by puromycin selection to establish stable cell lines. Cells expressing mito-Keima were maintained under standard culture conditions and used for subsequent experiments. Mitophagy was evaluated by flow cytometry and confocal microscopy.

### Co-immunoprecipitation

Following the manufacturer’s directions, RAW264.7 cells were lysed using RIPA buffer that included protease and phosphatase inhibitors. The cell lysates contained both cytoplasmic and membrane-associated proteins, so this approach allowed detection of their potential intracellular interactions. At 4 °C, the lysates were centrifuged at 12,000 × *g* for 15 min to separate the supernatants. For immunoprecipitation, equal amounts of protein were mixed with specific primary antibodies or control IgG and incubated overnight at 4 °C with gentle rotation, followed by a 2-h incubation with Protein A/G magnetic beads. The beads were thoroughly washed and then boiled in SDS loading buffer. Immunoprecipitated proteins were analyzed using Western blotting.

### Protein structure modeling

The amino acid sequences of ANGPTL2 (UniProt ID: Q9R045) and IGFBP5 (UniProt ID: Q07079) were obtained from the UniProtKB database (https://www.uniprot.org/). ANGPTL2 and IGFBP5 proteins were docked using HDOCK (http://hdock.phys.hust.edu.cn/) [90]. The predicted structures were visualized and analyzed with PyMOL (Schrödinger, New York, USA) to identify potential interaction domains. The structural quality was evaluated based on the docking scores.

### Intra-joint injection of pAAV-CMV-*Angptl2*

The coding sequence for Angptl2 was cloned into the pAAV-CMV-*Angptl2* vector, while the control sequence was inserted into the pAAV-CMV-EGFP vector to create adeno-associated virus (AAV) constructs. AAV-*Angptl2* and AAV-Ctrl were packaged and purified by JTS Scientific Inc. (Wuhan, China). Following the second immunization, mice were anesthetized with isoflurane every two days, and 5 μL of AAV-*Angptl2* (9.14 × 10¹² vgs/mL) or AAV-Ctrl (5.22 × 10¹² vgs/mL) was injected intra-articularly into the wrist and ankle joint cavities using a step-graded Hamilton syringe fitted with a 34-gauge needle. AAV transduction efficiency was verified through RT-qPCR and Western blot analysis.

### Statistical analysis

The results are representative of no fewer than three separate experiments. Data are displayed as mean ± SD. Statistical evaluations were carried out with GraphPad Prism 10 (GraphPad Software Inc., California, USA). Before analyzing, all the data have been under normal distribution test (Shapiro-Wilk test) and homogeneity of variance test (Brown-Forsythe test). A two-tailed unpaired Student’s *t* test was used for comparing two groups, while one-way ANOVA with Tukey’s post hoc test was applied for comparisons among three or more groups. Throughout the entire research process, the significance threshold was defined as *p < 0.05, **p < 0.01, ***p < 0.001, ****p < 0.0001. Investigators were blinded to group allocation during animal experiments, including treatment administration and outcome assessment. No formal statistical methods were applied a priori to predetermine sample size; instead, the number of animals per group was based on previous studies employing the CIA model, which established similar sample sizes as sufficient to detect meaningful differences in disease outcomes.

### Ethics statement

All animal procedures were performed in accordance with the ARRIVE 2.0 guidelines and approved by the Ethics Committee of The Hospital of Stomatology, Wuhan University (S07924110I).

## Supplementary information

Supplementary information

Original WB figure

## Data Availability

The datasets generated and/or analyzed during the current study can be obtained from the corresponding author upon reasonable request.
