# Supplementary material for: ANGPTL2 inhibits macrophage pyroptosis and alleviates rheumatoid arthritis progression by regulating mitophagy via IGFBP5
Source: Cell Death Dis. 2026 Mar 12;17(1):309. doi: 10.1038/s41419-026-08537-z (PMC13039533; doi:10.1038/s41419-026-08537-z)
Supplement: Supplementary file 1 — Supplementary information [file 41419_2026_8537_MOESM1_ESM.pdf]

SUPPLEMENTARY INFORMATION

ANGPTL2 Inhibits Macrophage Pyroptosis and Alleviates Rheumatoid Arthritis Progression by Regulating Mitophagy via IGFBP5

Yuqi Liu [a, †], Qiudong Yang [a, †], Zhendong Huang [a], Jiahui Sun [a], Junhong Xiao [a], Zhengkun Yang [a], Xin Huang [a, b], Li Ma [a, b], Xiaoxuan Wang [a, b], Chuan Wang [a, b], Zhengguo Cao [a, b, *]

[a] State Key Laboratory of Oral & Maxillofacial Reconstruction and Regeneration, Key Laboratory of Oral Biomedicine Ministry of Education, Hubei Key Laboratory of Stomatology, School & Hospital of Stomatology, Wuhan University, Wuhan, China

[b] Department of Periodontology, School & Hospital of Stomatology, Wuhan University, Wuhan, China

[†] These authors contributed equally to this work.

[*] Corresponding author at: Department of Periodontology, School & Hospital of Stomatology, Wuhan University, 237 Luoyu Road, Hongshan District, Wuhan 430079, China.

This PDF files includes:

Figures S1 to S6

Tables S1 to S2

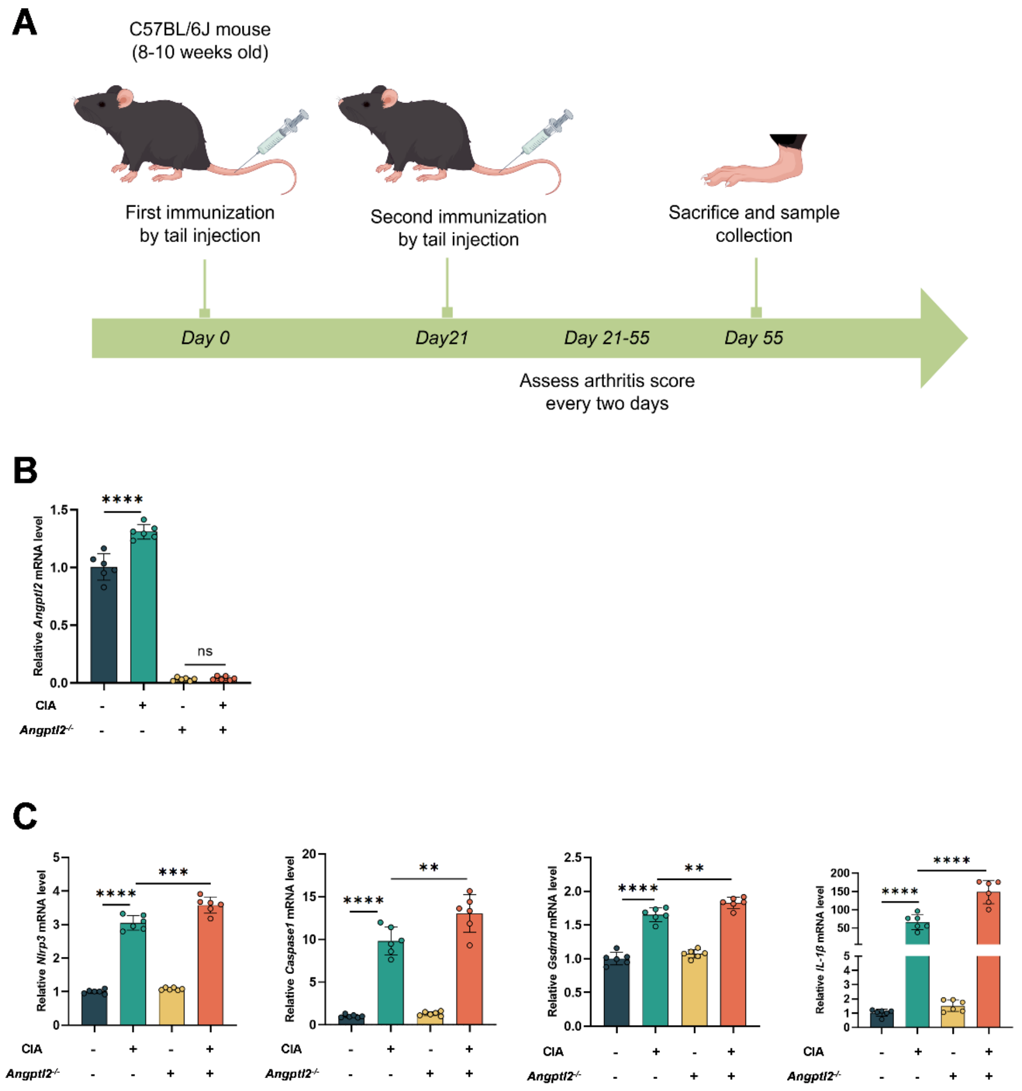

Figure S1. (A) Schematic diagram of the collagen-induced arthritis (CIA) model used in ANGPTL2-deficient (*Angptl2*$^{-/-}$) and wild-type (WT) mice. (B) qPCR analysis of *Angptl2* mRNA expression in joint tissues. (C) qPCR analysis of *Nlrp3*, *Caspase-1*, *Gsdmd*, and *Il-1β* mRNA expression in joint tissues. *$p < 0.05$, **$p < 0.01$, ***$p < 0.001$, ****$p < 0.0001$ in the indicated groups.

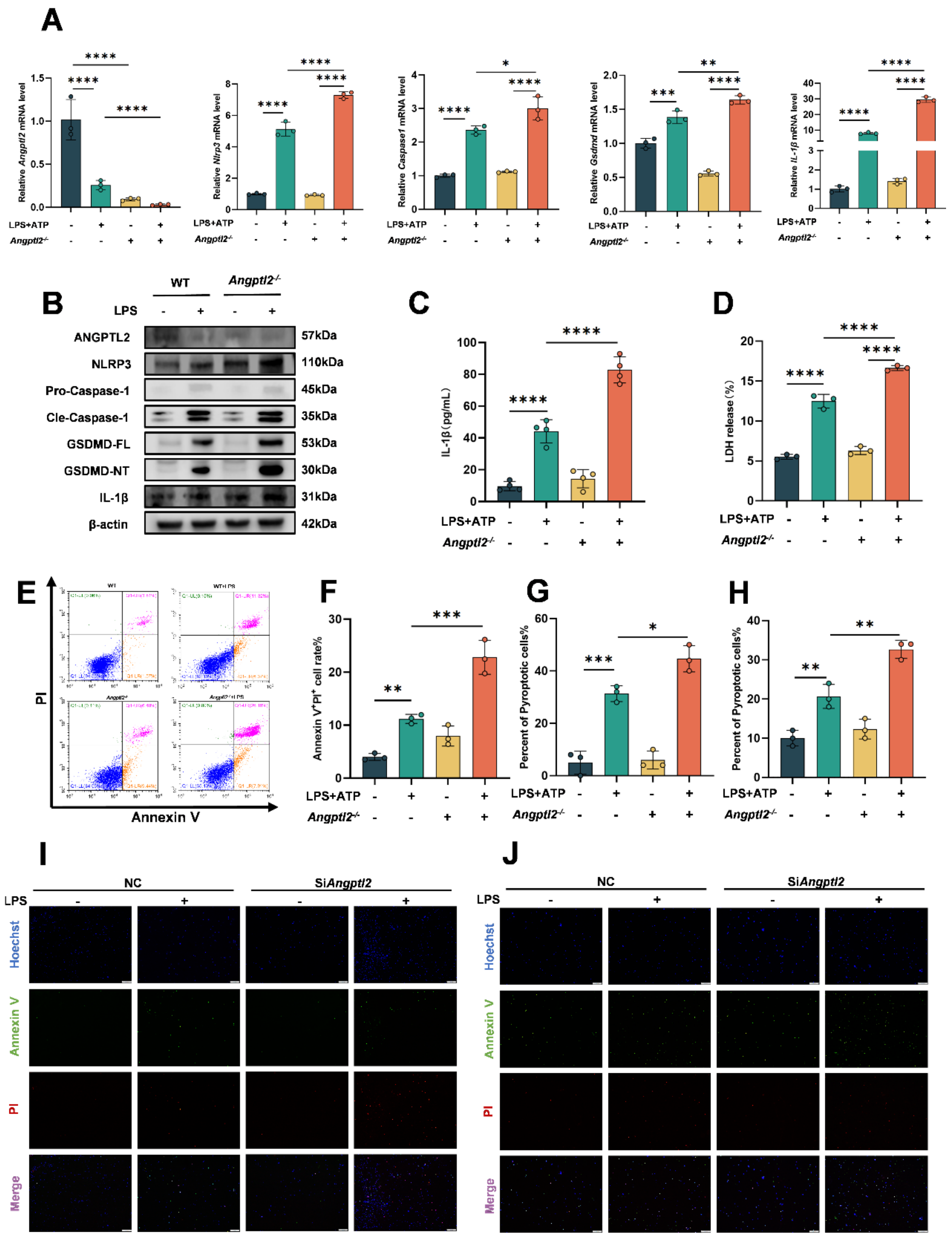

Figure S2. ANGPTL2 knockout increases pyroptosis-related gene and protein expression and inflammasome activation in primary macrophages. (A) qPCR analysis of *Angptl2*, *Nlrp3*, *Caspase-1*, *Gsdmd*, and *Il-1β* mRNA levels in BMDMs derived from wild-type (WT) and *Angptl2*$^{-/-}$ mice under basal and LPS + ATP-

stimulated conditions. (B) Western blot analysis of ANGPTL2, NLRP3, GSDMD, Caspase-1, and IL-1β proteins in BMDMs from WT and *Angptl2*$^{-/-}$ mice. (C) ELISA measurement of IL-1β levels in supernatants from WT and *Angptl2*$^{-/-}$ BMDMs. (D) LDH release assay assessing cell death in WT and *Angptl2*$^{-/-}$ BMDMs. (E) Representative flow cytometry plots showing Annexin V and PI staining of WT and *Angptl2*$^{-/-}$ BMDMs. (F) Quantification of Annexin V^{+}PI^{+} cell populations shown in (E). (G-H) Quantification of the percentage of pyroptotic cells in RAW 264.7 cells (G) and BMDMs (H) observed under TEM. (I-J) Representative fluorescence microscopy images of RAW264.7 cells(I) or BMDMs(J) stained with Annexin V and PI under indicated conditions. *p < 0.05, **p < 0.01, ***p < 0.001, ****p < 0.0001 in the indicated groups.

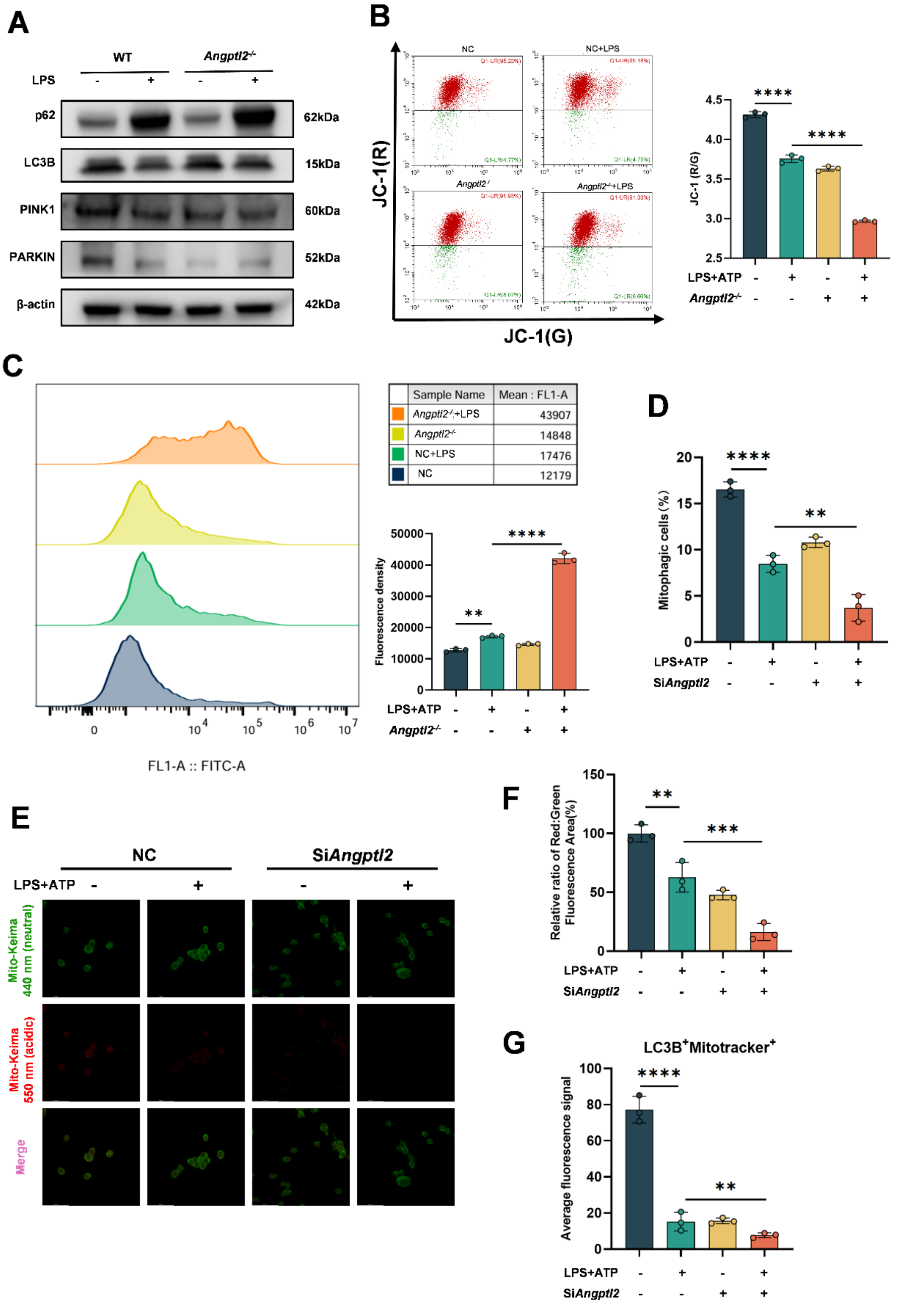

Figure S3. Evaluation of mitochondrial damage and mitophagy in ANGPTL2-deficient macrophages. (A) Western blot analysis of mitophagy-related proteins (PINK1, PARKIN, LC3B, and p62) in BMDMs from WT and *Angptl2*$^{-/-}$ mice, with or without LPS + ATP stimulation. (B) Flow cytometry analysis of mitochondrial

membrane potential in WT and *Angptl2*$^{-/-}$ BMDMs using JC-1 staining. The red-to-green fluorescence ratio (R/G) was used to assess mitochondrial polarization. (C) Flow cytometry analysis of intracellular total ROS levels in WT and *Angptl2*$^{-/-}$ BMDMs under the same stimulation conditions. (D) Quantification of the percentage of mitophagic cells in each sample using flow cytometry. (E) Representative confocal microscopy images of RAW264.7 cells expressing mito-Keima, showing mitochondrial localization in neutral (green) and acidic (red) environments. (F) The relative mean mt-Keima 550/440 ratio was quantified by normalizing to NC group in RAW 264.7 cells. (G) Quantification of LC3B-MitoTracker colocalized signal intensity. *$p < 0.05$, **$p < 0.01$, ***$p < 0.001$, ****$p < 0.0001$ in the indicated groups.

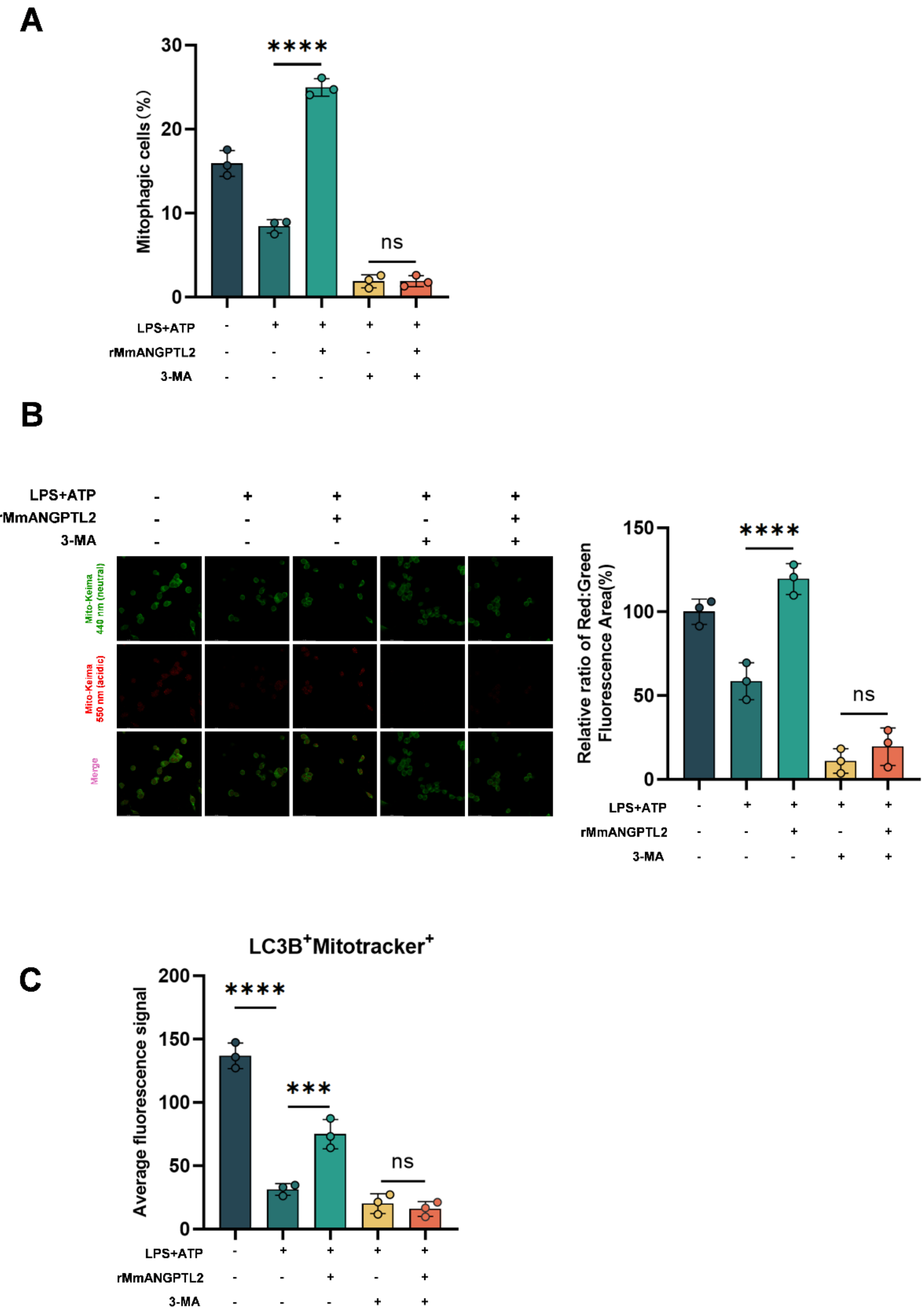

Figure S4. Evaluation of mitophagy in rMmANGPTL2 macrophages. (A) Quantification of the percentage of mitophagic cells in each sample using flow cytometry. (B) Representative confocal microscopy images of RAW264.7 cells expressing mito-Keima, showing mitochondrial localization in neutral (green) and

acidic (red) environments. The relative mean mt-Keima 550/440 ratio was quantified by normalizing to NC group in RAW 264.7 cells. (C) Quantification of LC3B-MitoTracker colocalized signal intensity. $*p < 0.05$, $**p < 0.01$, $***p < 0.001$, $****p < 0.0001$ in the indicated groups.

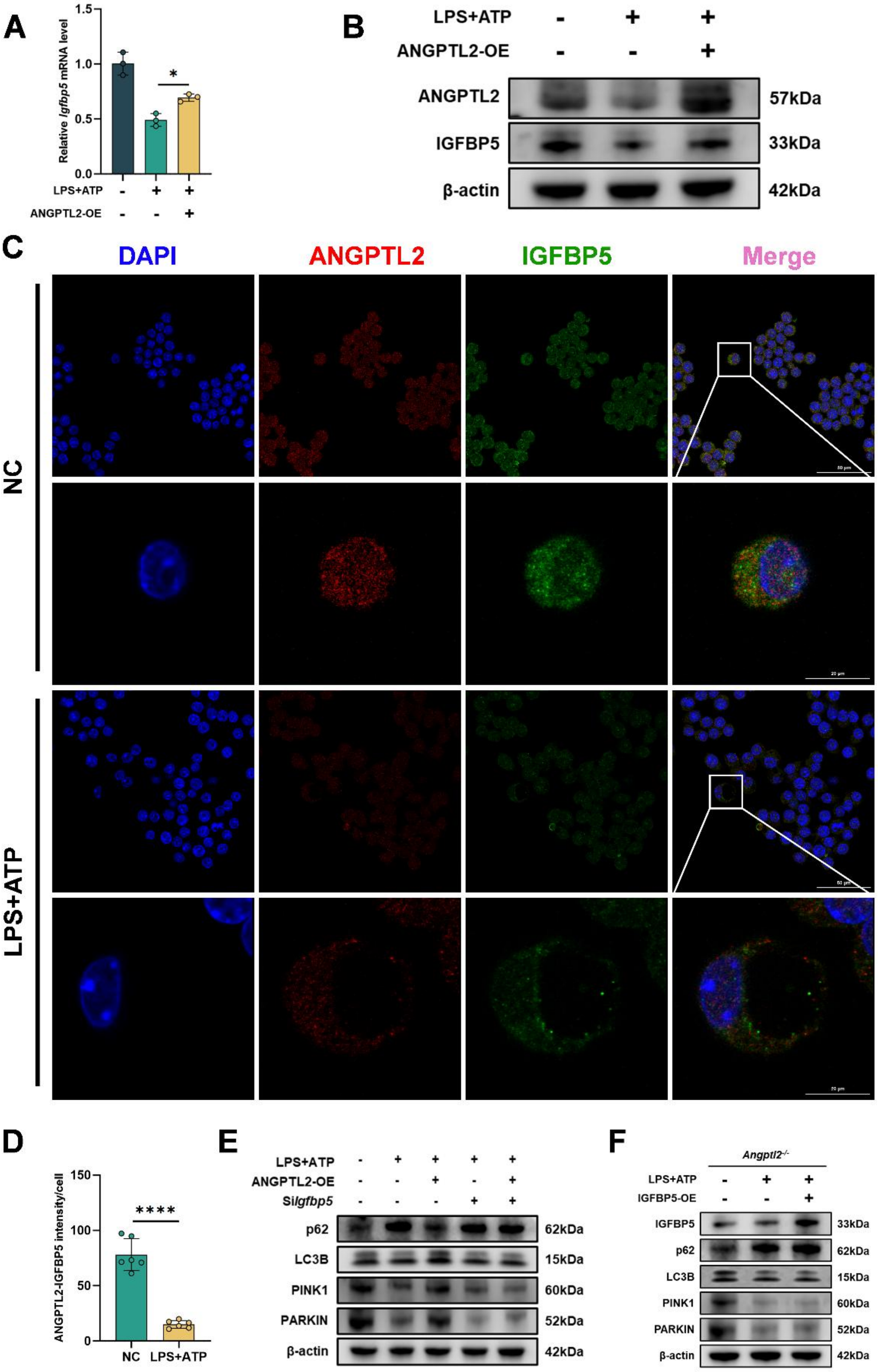

Figure S5. ANGPTL2 forms a protein complex with IGFBP5 within the cell to regulate mitophagy. (A) qPCR analysis of IGFBP5 mRNA expression in BMDMs following ANGPTL2 overexpression and LPS + ATP treatment. (B) Western blot analysis of ANGPTL2 and IGFBP5 protein levels in BMDMs following ANGPTL2

overexpression and LPS + ATP treatment. (C) Confocal microscopy images showing colocalization of ANGPTL2 (red) and IGFBP5 (green) in RAW264.7 cells. (D) Quantification of ANGPTL2-IGFBP5 colocalized signal intensity per cell. (E) Western blot analysis of mitophagy-related proteins in BMDMs treated with LPS + ATP and ANGPTL2 overexpression, with or without IGFBP5 knockdown. (F) Western blot analysis of ANGPTL2 and IGFBP5 protein levels in BMDMs following IGFBP5 overexpression and LPS + ATP treatment. *p < 0.05, ****p < 0.0001 in the indicated groups.

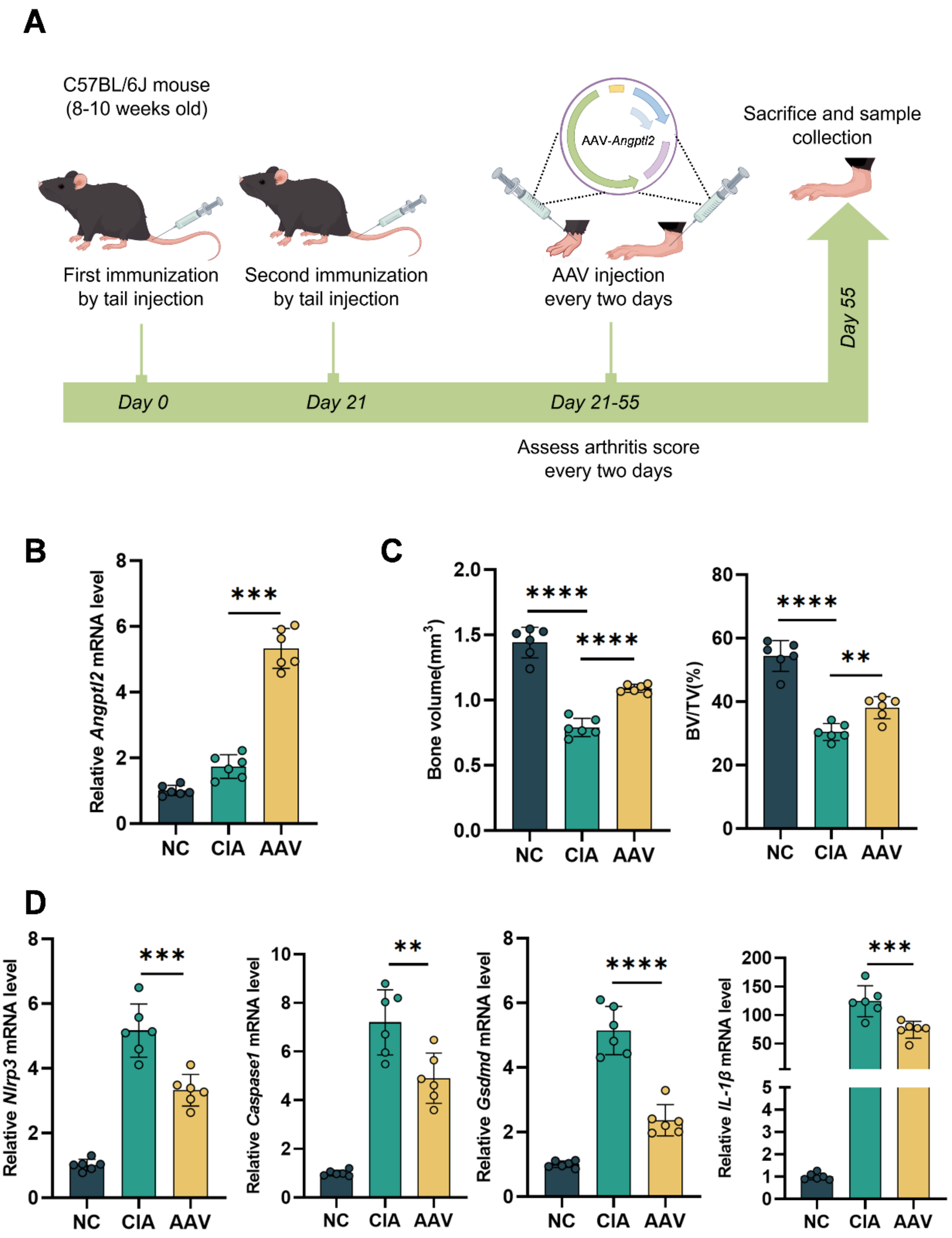

Figure S6. Validation of intra-articular ANGPTL2 delivery and its effects on bone and inflammation in CIA mice. (A) Diagram of the experimental timeline showing CIA model induction and intra-articular injection of AAV-*Angptl2*. (B) qPCR analysis of *Angptl2* mRNA expression in joint tissues to confirm transgene expression following

AAV delivery. (C) Quantification of talus bone volume (mm^3) and bone volume to tissue volume ratio (BV/TV, %) in ankle joints from control, CIA, and AAV-*Angptl2*-treated groups. (D) qPCR analysis of inflammasome- and pyroptosis-associated genes (*Nlrp3*, *Caspase-1*, *Gsdmd*, *Il-1β*) in ankle joints across experimental groups. *$p < 0.05$, **$p < 0.01$, ***$p < 0.001$, ****$p < 0.0001$ in the indicated groups.

Table S1

The sequences of primers used.

Name	F/R	Sequences
β-actin	F	GTGACGTTGACATCCGTAAAGA
	R	GCCGGACTCATCGTACTCC
Angptl2	F	CCACCTCGGGTCTACCAAC
	R	CTTGCAGGCAGTCTCTCCAT
Nlrp3	F	ATTACCCGCCCGAGAAAGG
	R	TCGCAGCAAAGATCCACACAG
Caspase-1	F	ACAAGGCACGGGACCTATG
	R	TCCCAGTCAGTCCTGGAAATG
Gsdmd	F	TTCAGGCCCTACTGCCTTCT
	R	GTTGACACATGAATAACGGGGTT
Il-1β	F	TTCAGGCAGGCAGTATCACTC
	R	GAAGGTCCACGGGAAAGACAC
Pink1	F	GCCTCATCGAGGAAAAACAGG
	R	GTCTCGTGTCCAACGGGTC
Parkin	F	GTGTTTGTCAGGTTCAACTCCA
	R	GAAAATCACACGCAACTGGTC
Igfbp5	F	CCCAATTGTGACCGCAAAGG
	R	GGCAGCTTCATCCCGTACTT

Table S2

The sequences for gene knockdown.

Name	Sequences
Si-*Angptl2*	CAGAGUCUUCCAAUCAGUUAATT
Si-*Igfbp5*	GAGAUGAGACAGGAAUCCGAATT
