# Supplementary material for: ANGPTL2 inhibits macrophage pyroptosis and alleviates rheumatoid arthritis progression by regulating mitophagy via IGFBP5
Source: Cell Death Dis. 2026 Mar 12;17(1):309. doi: 10.1038/s41419-026-08537-z (PMC13039533; doi:10.1038/s41419-026-08537-z)

Figure 1J

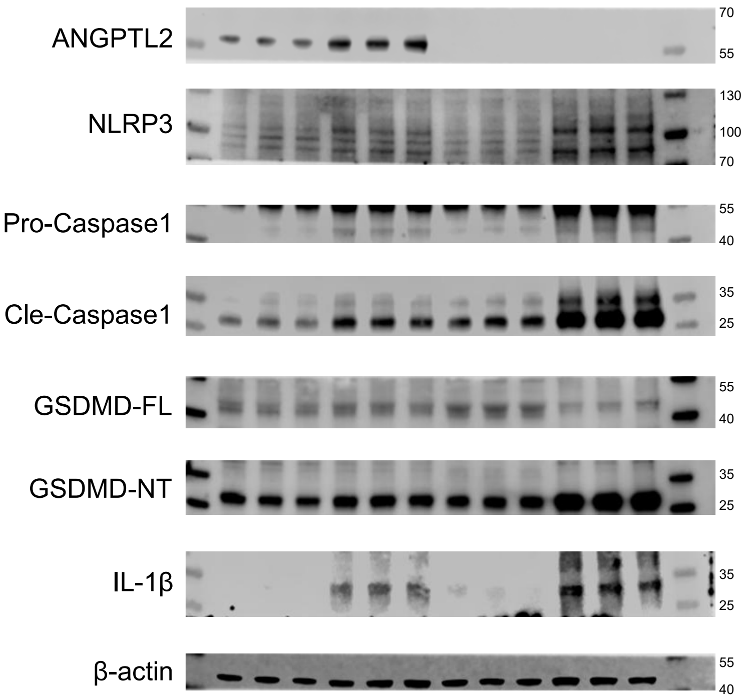

Figure 2D

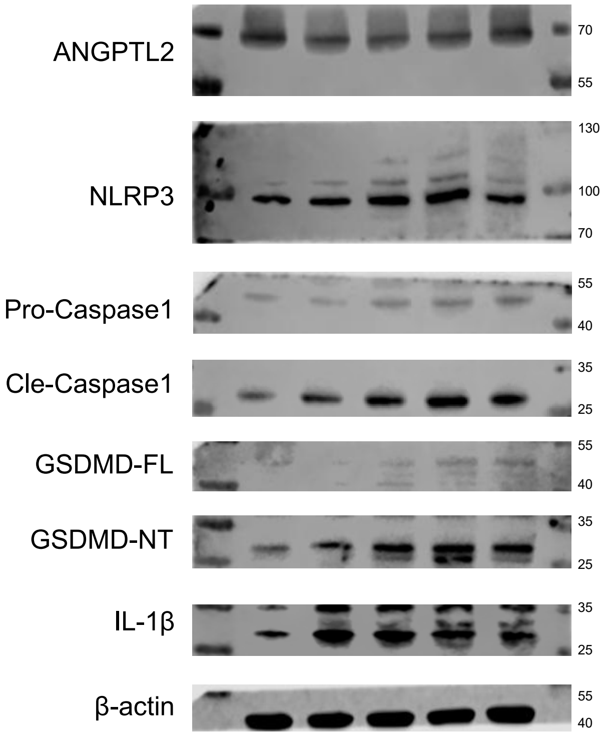

Figure 2H

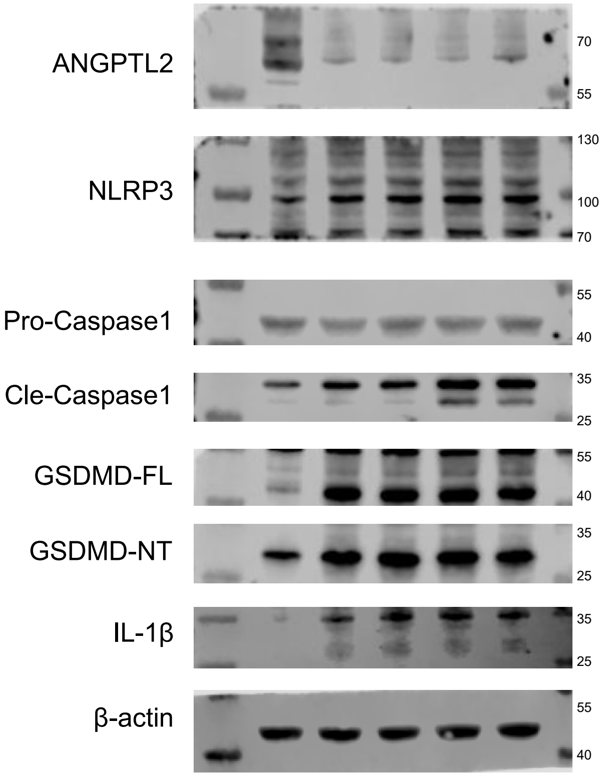

Figure 3C

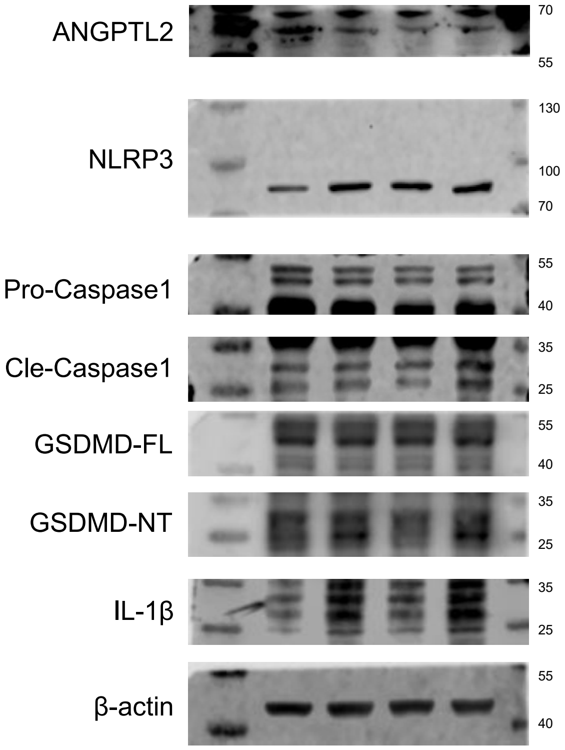

Figure 3D

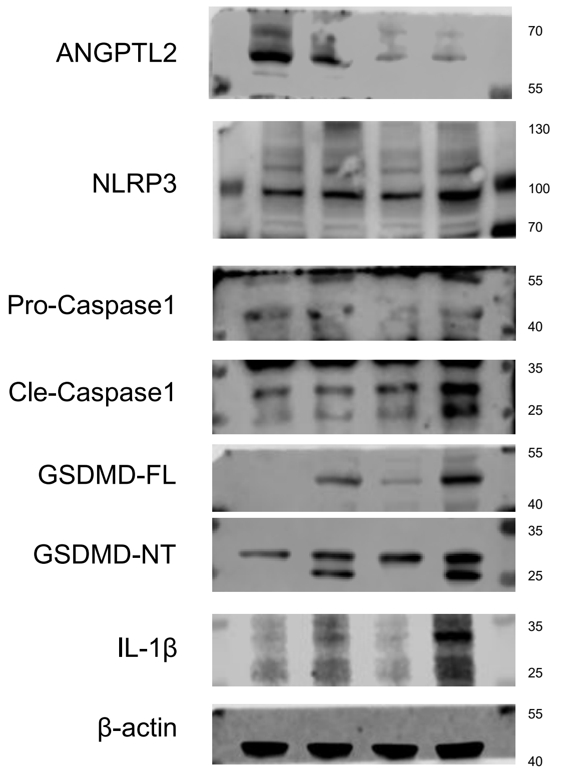

Figure S2B

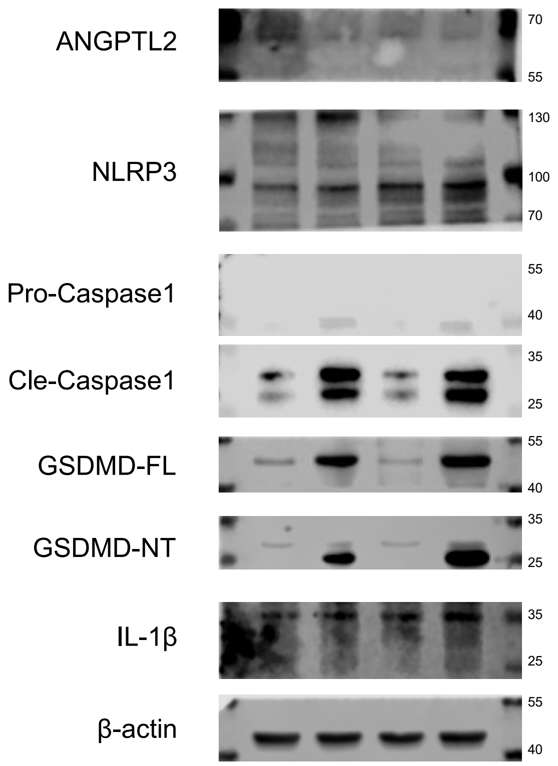

Figure 5A

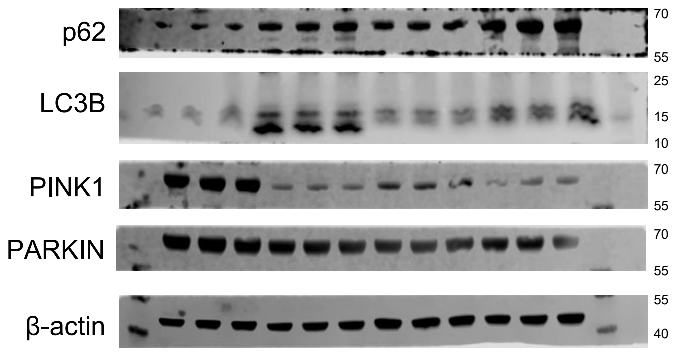

Figure 5B (LEFT)

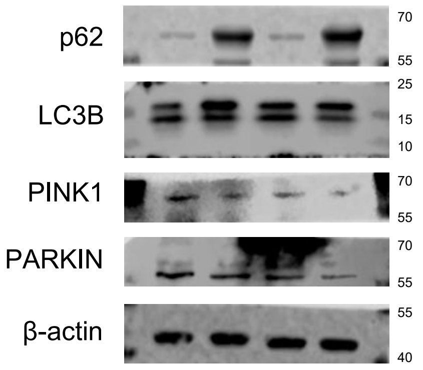

Figure 5B (RIGHT)

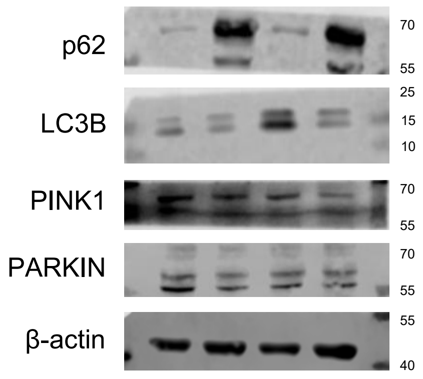

Figure S3A

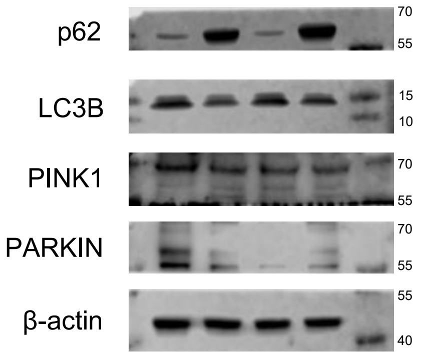

Figure 6A

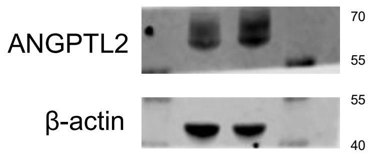

Figure 6F

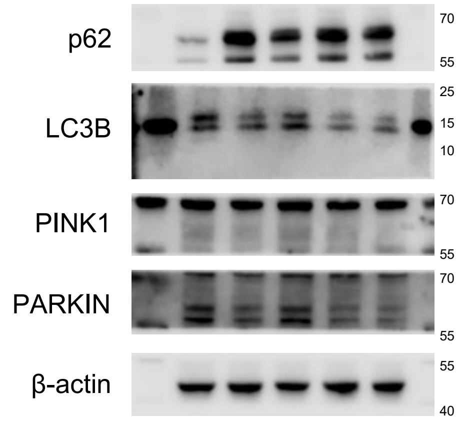

Figure 6H

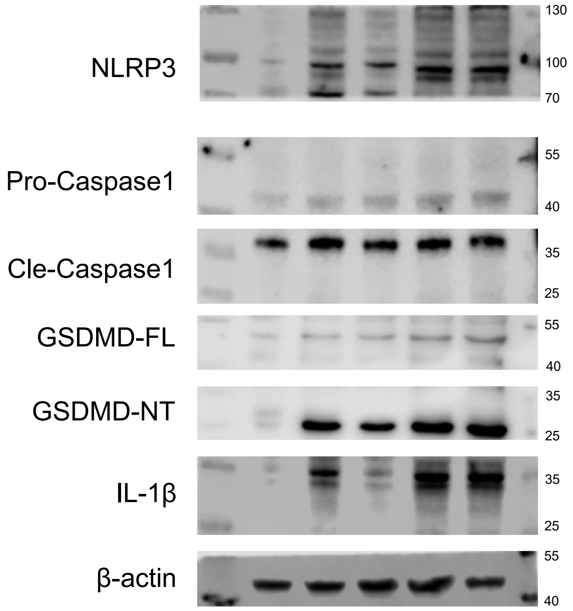

Figure 7C

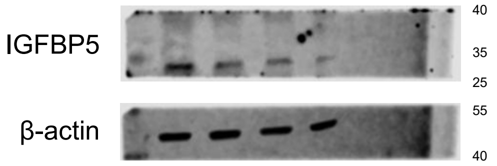

Figure 7D

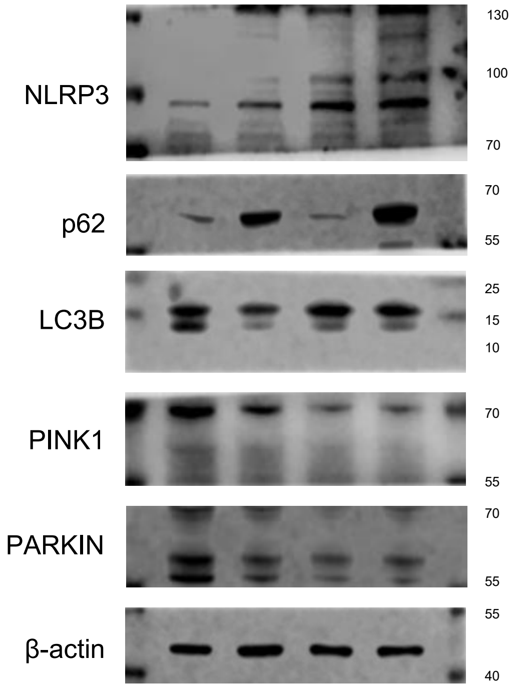

Figure 7F

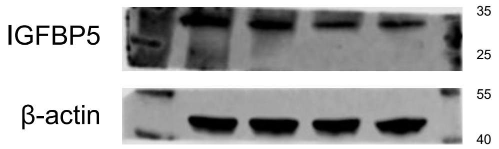

Figure 7H

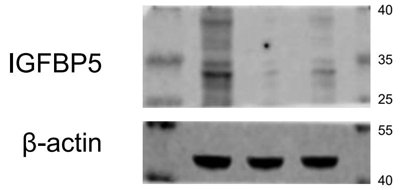

Figure 7I

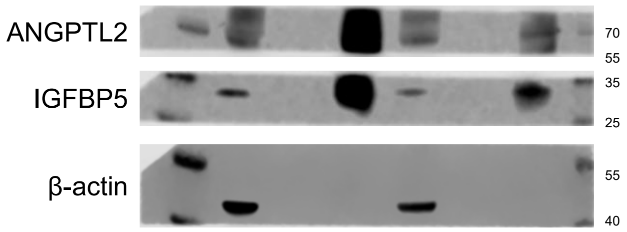

Figure 7J

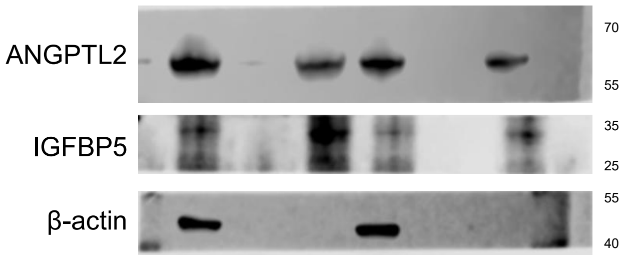

Figure 7L

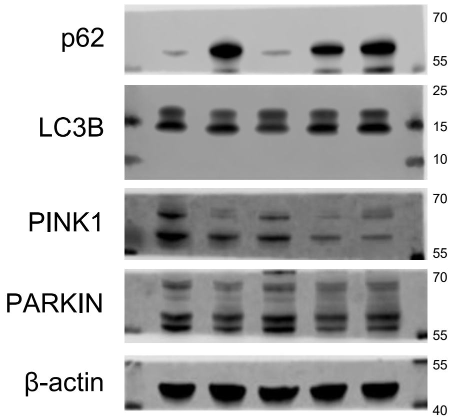

Figure S5B

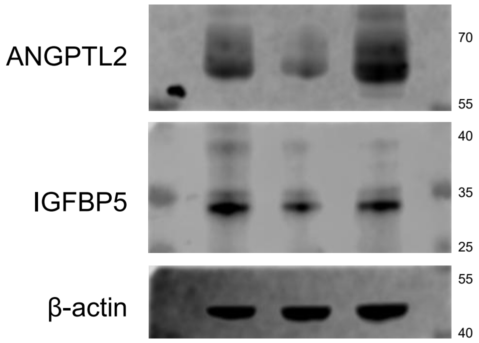

Figure S5E

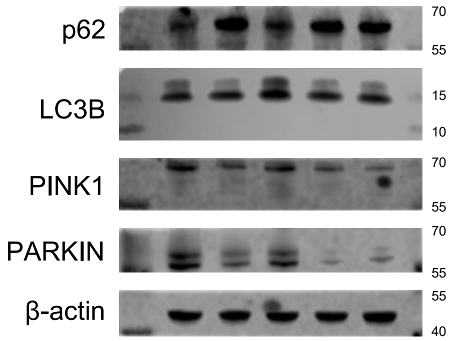

Figure S5F

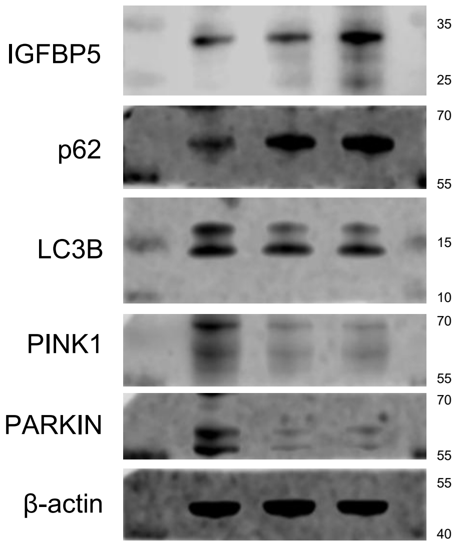

Figure 8I

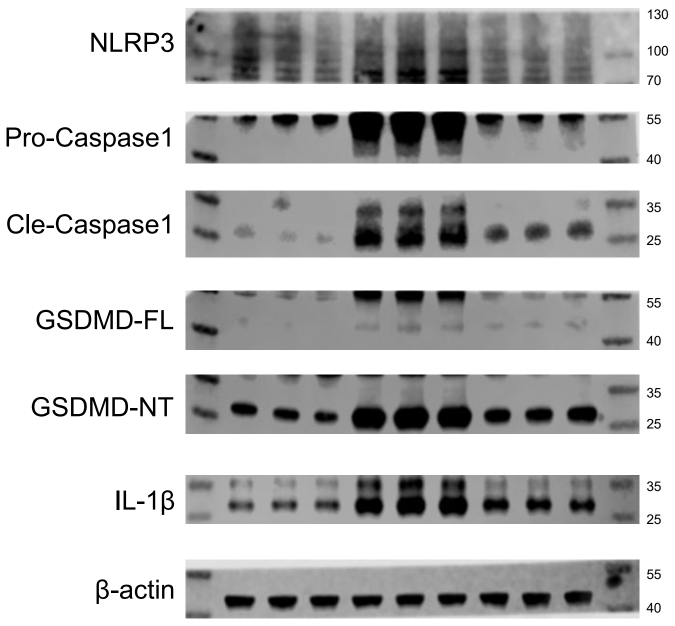

Figure 8H

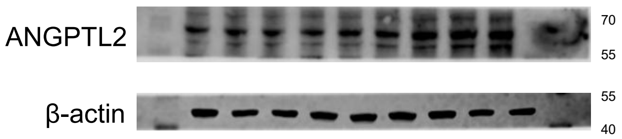

Figure 8J

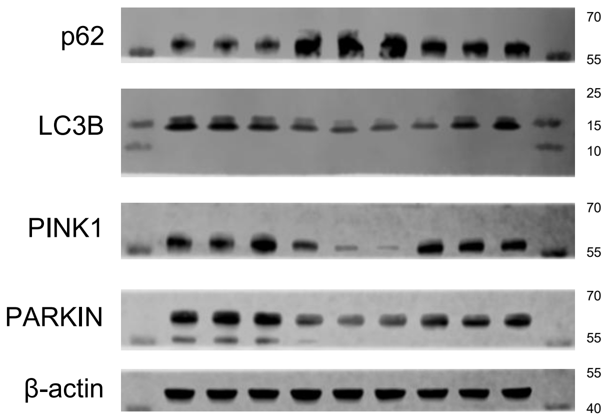

Supplement: Supplementary file 2 — Original WB figure [file 41419_2026_8537_MOESM2_ESM.pdf]
